# Supplementary material for: Acupuncture, counselling or usual care for depression and comorbid pain: secondary analysis of a randomised controlled trial
Source: BMJ Open. 2014 May 2;4(5):e004964. doi: 10.1136/bmjopen-2014-004964 (PMC4024599; doi:10.1136/bmjopen-2014-004964)
Supplement: Author's manuscript [file bmjopen-2014-004964.draft_revisions.pdf]

ACUPUNCTURE, COUNSELLING OR USUAL CARE FOR DEPRESSION AND COMORBID PAIN: SECONDARY ANALYSIS OF A RANDOMISED CONTROLLED TRIAL

Journal:	*BMJ Open*
Manuscript ID:	bmjopen-2014-004964
Article Type:	Research
Date Submitted by the Author:	30-Jan-2014
Complete List of Authors:	Hopton, Ann; University of York, Health Sciences<br>MacPherson, Hugh; University of York, Health Sciences<br>Keding, Ada; University of York, Health Sciences<br>Morley, Stephen; University of York, Health Sciences
**Primary Subject Heading**:	Complementary medicine
Secondary Subject Heading:	Mental health, General practice / Family practice, Evidence based practice
Keywords:	Depression, Comorbid pain, Acupuncture, Counselling, Usual Care

SCHOLARONE™
Manuscripts

For peer review only

ACUPUNCTURE, COUNSELLING OR USUAL CARE FOR DEPRESSION AND COMORBID PAIN: SECONDARY ANALYSIS OF A RANDOMISED CONTROLLED TRIAL

For peer review only

A. Hopton[1], H. MacPherson[1], A. Keding [1], S. Morley[2]

[1] Department of Health Sciences, University of York, UK

[2]Leeds Institute of Health Sciences - University of Leeds

Address for correspondence:

Ann Hopton
Research Fellow
Department of Health Sciences
University of York
Heslington
York YO10 5DD
United Kingdom
Tel: 01904 321384
E-mail: ann.hopton@york.ac.uk

Keywords: Depression, comorbid pain, acupuncture, counselling, usual care
Words 3,708.

For peer review only - http://bmjopen.bmj.com/site/about/guidelines.xhtml

ABSTRACT

Background

Depression with comorbid pain is associated with a poor response to various types of treatment. The objective in this study is to determine whether patients reporting pain have different depression and pain outcomes over time in response to acupuncture, counselling or usual care.

Methods

Self-reported ratings of depression and pain from 755 patients in a pragmatic randomised controlled trial of acupuncture (302) or counselling (302) compared to usual care alone (151) are described and analysed using a series of regression models and ANCOVA. Patients reported outcomes of PHQ-9 for depression, SF36 bodily pain and EQ-5D, all at baseline, 3, 6, 9, and 12 months.

Results

At baseline, 755 patients reported EQ-5D pain categories; 384 (50.9%) reported moderate to extreme pain. Controlling for baseline depression, a linear regression model showed that the presence of pain at baseline was associated with poorer depression outcomes at 3 months mean difference = 1.16, (95%CI = 0.12 to 2.2) Participants with moderate to extreme pain at baseline did better at 3 months if they received acupuncture (mean reduction in PHQ-9 from baseline = 6.0, 95%CI = 5.0 to 7.1 and a mean reduction in SF-36 bodily pain = 11.2, 95% CI= 7.1 to 15.2 compared to improvements for those who received counselling (4.3, 95%CI = 3.3 to 5.4; 7.6, 95% CI = 3.6 to 11.6) or usual care (2.7, 95%CI = 1.50 to 4.0: 7.2, 95% CI= 2.3 to 12.1). In comparison, no notable differences were seen between treatment arms within the no pain comparator group.

Conclusion

Patients with depression and pain at baseline recovered less well from treatment over three months than those with depression and no pain. Reductions in both depression and pain were most marked in the acupuncture group, followed by the counselling group, and then the usual care group.

(300 words)

Strengths:

- This study targets the under researched area of how pain may impact on the outcome of different treatments for depression.
- The research questions were clearly defined to establish the prevalence of pain in a depressed population, to describe the demographic profile of depressed people in pain compared to those who are depressed and pain free, to determine the impact of pain on the treatment outcomes, and in turn determine the effect of treatment for depression on the level of pain reported in the months subsequent to receiving treatment for depression.
- As a sub study of a larger pragmatic trial, the findings are important for three reasons; firstly the emphasis on external validity with findings that are generalizable to primary care patients with depression across the UK. The method of randomisation to treatment allocation is a feature that was maintained through the sub-grouping into a ‘pain group’ and ‘no pain group’ to provide control for temporal effects and random factors that might influence the outcome of treatment.
- The standardised treatment protocols allowed for treatment to be tailored to individual patient’s needs regarding pain and depression whilst maintaining high standards of care, qualifications and practice experience. In terms of limitations,

Limitations:

- This study was a sub study of a larger trial which was not powered to detect differences between the subgroups of moderate to extreme pain and no pain.
- The use of the BDI-II outcome was not measured at 3 months.
- Changes to factors such as sleeplessness, energy loss from within the BDI-II could not be assessed; however, The medication prescribed to participants and the frequency of use was not recorded at baseline or as follow up data,
- The impact of the treatment on the types of anti-depressant and analgesic medication used or the frequency of use could not be established.

For peer review only

INTRODUCTION

Around 50%-66% of patients who are depressed also report pain.[1,2] Depression accentuates existing pain problems[3] adding to the emotional distress and poor sleep irrespective of whether the pain had a known cause[4]. Increased pain symptoms may also represent a greater severity of depression rather than a poor prognostic factor.[5] In terms of treatment, comorbid pain and depression are present in two thirds of depressed primary care patients who commence antidepressant therapy.[6] Pain is known to interfere with the routine activities in 42% of primary care patients prior to starting pharmacological treatment for depression[7] and has a strong negative impact on achieving remission.[8] Too frequently there is a focus on the treatment of either pain or depression. Treatment outcomes may be enhanced by taking an holistic approach, and delivering treatment options that address both pain and depression together.[9]

A meta-analysis of 18,000 participants data from 25 high quality trials has shown that acupuncture is an effective treatment option for several chronic pain conditions.[10] Acupuncture is also regularly provided by acupuncturists to treat depression[11] although not routinely available as a treatment option for depression within the UK's National Health Service.[12] Counselling for depression is widely available in primary care practices[13] however there is limited evidence for counselling compared to usual care as a treatment for patients with depression and a chronic physical health problem.[4,14] A recent randomised controlled trial of acupuncture or counselling provided to patients with depression found that both interventions significantly reduced depression at three months when compared to usual care.[15] However, the published trial data did not compare the treatment outcomes of patients who had comorbid pain and depression with those who had depression alone.

The primary aim of this study is to find out whether people with depression who are also in pain have better or worse depression outcomes than those without pain. In terms of objectives, we document the prevalence of pain in a depressed population, the demographic profile of patients in pain and the relationship between pain and depression. We then determine depression and pain outcomes following treatment with acupuncture or counselling when compared to usual care alone.

METHODS

Design

This research is a sub-study nested within a three-arm randomised controlled trial of acupuncture or counselling as adjuncts to usual care compared to usual care alone.[15] Full details of the ACUDep trial protocol are reported elsewhere.[16] Whilst maintaining the integrity of the 2:2:1 randomisation allocation of the ACUDep trial[15], for the purpose of this sub-study participants were divided into two groups according to their response to the EQ-5D pain statements at baseline: I have no pain or discomfort; I have moderate pain or discomfort; I have extreme pain or discomfort. People who reported either moderate or extreme pain were considered together as the "pain group", the remainder were assigned as a "no pain comparator group". A summary of the sub-group allocations and other demographic variables are presented in Table 1.

Participants:

Recruited to the ACUDep trial[15] were 755 patients aged 18 or over and diagnosed with depression or who had consulted for depression within the previous 30 months with a baseline score of 20 or above on the Beck Depression Inventory-II, which this scale classes as "moderate" to "severe" depression.[17] Patients were recruited from general practices in rural and urban areas of Yorkshire, County Durham and Northumberland. Bodily pain scores on the SF-36[18] and any chronic physical conditions or pre-existing illnesses were reported by patients at baseline. Patients' details were recorded by the York Trials Unit and randomised by computer-generated block randomisation, with block sizes of 5 and 10 by an investigator with no clinical attachment to the trial. An unequal allocation ratio of 2:2:1 to acupuncture, counselling and usual care provided groups of 302, 302 and 151 respectively.

Interventions:

Within the main ACUdep trial[15] participants allocated to the acupuncture and counselling groups received the offer of up to 12 sessions on a weekly basis. The acupuncture intervention was performed according to a treatment protocol developed and agreed by acupuncture practitioners, which allowed for individualised treatment within a standardised theory-driven framework. The acupuncturists were members of the British

For peer review only

Acupuncture Council with a minimum post qualification experience of three years, specific details of the intervention are published elsewhere[19] The treatment protocol for the counselling intervention was based on competences independently developed for Skills for Health within National Occupational Standards for Psychological Therapies.[20] The counsellors were members of the British Association for Counselling and Psychotherapy with a minimum of 400 hours post qualification experience and used a non-directive approach to help clients express feelings, clarify thoughts, and reframe difficulties. Usual care available to all participants throughout the trial included prescribed pharmaceutical and other interventions provided by NHS primary or secondary mental health services.

Outcome measures

The outcome measure used for this sub-study were collected as part of the ACUDep trial[15], comprising the primary depression score as measured by the Patient Health Questionnaire (PHQ-9)[21], which scores each of the 9 DSM-IV criteria for depression as “0” (not at all) to “3” (nearly every day). The primary endpoint was at 3 months, with further follow up at 6, 9 and 12 months. The patients’ experience of the effect of the treatment received can also be expressed as depression free days[22] which is an approximate summary measure derived from the PHQ-9 cut off scores averaged over the period between measurements[15]. Patient reported pain measures were collected at baseline and at 3, 6, 9, and 12 months follow up using the SF-36 Bodily Pain sub-scale, which has a range 0-100, where a score of 100 indicated no pain, and the EQ-5D categories of no pain or moderate pain or severe pain. At baseline, patients also recorded their use of anti-depressants and analgesic medication, and their preferences and expectations of treatment.

Data analysis

Objective 1

A descriptive analysis of the demographic profile of the pain group and no pain comparator group was conducted, and the participants’ gender, average age of the sample population and pain groups population, their age on leaving education, the onset of their depression, the number of episodes they experienced, and the baseline scores of PHQ-9 and BDI-II baseline, SF-36 bodily pain score reported.(Table1) ANOVA was used to compare the mean baseline PHQ-9 scores between the pain group and no pain comparator group. For the pain group alone, a Kendall’s Tau correlation was used to test the association between the baseline scores of the PHQ-9 and SF-36 bodily pain score. This test was considered the most appropriate given the possibility of tied scores between the PHQ-9 and SF-36 bodily pain.

Objective 2

A series of regression models was applied to determine the influence of baseline pain on depression outcome at 3 months. The first model predicted PHQ-9 at 3 months from the baseline EQ-5D Pain Group (‘pain’ or ‘no pain’), controlling for baseline PHQ-9 scores. Systematic univariate analysis of demographic variables, the BDI-II depression items and the five EQ-5D items, whilst controlling for PHQ-9 scores at baseline with the level of significance set at $p<0.1$ was conducted to identify potential predictors of PHQ-9 depression at 3 months. The variables identified were then included in a final linear regression model ($p<0.05$).

Using any remaining significant predictors and controlling for baseline PHQ-9 depression scores ANCOVA was used to test whether baseline pain affected treatment outcomes measured by the PHQ-9 score at three months. An interaction term between treatment and pain group in this model was used to establish whether patients in pain responded differently with regards to their depression to the treatments than patients reporting no pain. Also using any remaining significant predictors and controlling for baseline PHQ-9 depression scores ANCOVA was used to test whether baseline pain affected treatment outcomes measured by the number of depression free days at three months; an approximate summary measure derived from PHQ-9 cut-off scores averaged over the period between measurements.[15,23]

An ANCOVA model controlling for controlling for baseline depression and baseline SF-36 bodily pain was used to assess the impact of treatment for depression on the bodily pain scores at 3 months. Descriptive analysis of the depression and pain scores was conducted over the 12 month follow up period. Finally a comparison of the adverse events reported between baseline and 12 month follow up was conducted using proportions and odds ratio.

RESULTS

Objective 1: An exploration of pain in a depressed population

What is the prevalence of pain?

For peer review only

Patients reported pain or discomfort on the EQ-5D questionnaire at baseline; 371 (49.1%) reported no pain or discomfort, 298 (39.5%) reported moderate pain or discomfort and 86 (11.4%) reported extreme pain or discomfort. The distribution of SF-36 bodily pain scores was negatively skewed; 140 (18.5%) patients reported a score of 100, indicating no pain at all, and the sample returned a median pain score of 52 (Interquartile 1= 25, Interquartile 3 = 84).

Allocation to "pain" and "no-pain" sub-groups

For the purpose of this study, those reporting moderate and extreme pain or discomfort on the EQ-5D questionnaire were merged together to form a single 'pain group' (n=384, 51%) with the remainder forming the 'no pain' comparator group (n=371, 49%). Patients who omitted to answer the EQ-5D pain question at baseline (n=7; 1%) were assigned to a group according to their SF-36 bodily pain score; 3 scored above the 3rd interquartile range of the SF-36 bodily pain scale and were assigned to the no pain group, the remaining four patients at baseline scored below the median and were assigned to the pain group. Within the defined pain and no-pain subgroups, trial arm allocations retained the 2:2:1 ratio of the original ACUDep[15] randomisation process.

What is the demographic profile of patients in pain and not in pain?

A summary of the demographic profiles, presented in Table 1, show that the pain and the no-pain groups were comparable for most baseline variables. Notable exceptions were: The pain group members tended to be older than the no pain comparator group (mean of 47 years, vs. 40 years) a difference which remained notable after temporarily removing ten pain group members with outlying ages between of 76-93 years. In terms of health and employment, 56% of the pain group (vs. 9% of the no-pain group) reported a painful health condition or illness that predated the onset of depression, for which 64% (vs. 31%) used analgesic medication regularly, 32% (vs. 9%) were unable to work or retired.

What is the relationship between pain and depression at baseline?

The baseline PHQ-9 depression scores indicate that the pain group reported higher levels of depression at baseline (mean PHQ-9=17.0, SD 5.2) than the no pain comparator group (mean PHQ-9=14.9, SD 5.2). Results of ANOVA confirmed the difference to be highly significant (mean difference 2.02, 95%CI =1.28 to 2.76). For the pain group alone the correlation between the PHQ-9 scores and SF-36 bodily pain scores was weak, but highly significant (Kendall's Tau -0.172, p<0.001).

Table 1. Demographics and variables of interest

Characteristic	No Pain N= 371 (49%)	Moderate-extreme pain N= 384 (51%)	Total N=755 (100%)
Age			
Mean (SD)	39.9 (11.58)	46.83 (14.11)	43.5 (13.37)
Median (min, max)	39 (18,75)	46 (18,93)	43 (18,93)
IQR (25%, 75%)	31,48	38,56	33,53
Missing	-	-	-
Sex			
Male	88 (23.7%)	113(29.4%)	201 (26.6%)
Female	283 (76.3%)	271(70.6%)	554(73.4%)
Missing	-	-	-
Age left education			
Mean (SD)	18.3 (3.90)	17.53 (4.61)	18.0 (4.37)
Median (min, max)	17.0 (13, 52)	16 (14, 54)	16 (13-54)
IQR (25%, 75%)	16, 21	16,18	16,19
Missing	-	-	-
Employment			
Full-time education	13 (3.5%)	10 (2.6%)	23 (3.1%)
Working full-time	167 (45%)	114 (29.7%)	281 (37%)
Working part-time	83 (22.4%)	61 (15.9%)	144 (19.5%)
Unable to work	19 (5.1%)	76 (19.8%)	95 (12.9%)
Looking after home	41 (11.1%)	42 (10.9%)	83 (11%)
Retired	14 (3.8%)	51 (13.3%)	65(8.8%)
Other	26 (7%)	22 (5.7%)	48(6.5%)
Missing	8 (2.1 %)	8 (2.1%)	16(2.1%)
Painful health or medical condition			
Current painful health or medical condition	41(12.7%)	299 (77.9%)	346 (45.8%)
Onset before depression	32(8.6%)	216 (56.2%)	248 (32.8%)

Missing	2(0.5%)	4(1%)	6 (0.8%)
Type of health problem			
Musculoskeletal	21(6%)	221(58%)	242(32%)
Other	27(7%)	81 (21%)	108(14%)
No health problem	321(87%)	81 (21%	402 (53%)
SF-36 Bodily pain			
Mean (SD)	76.97(22.53)	39.03(22.5)	57.6 (28.44)
Median (min, max)	77.5 (12, 100)	41 (0, 100)	52 (0-100)
IQR (25%, 75%)	62, 100	22, 51	32, 84
Missing	3 (0.39%)	1 (1.3%)	4 (0.5%)
Depression			
In last 2 weeks	264 (71.2%)	310 (80.7%)	574 (76.1%)
Missing	2 (0.5%)	10 (2.6%)	12 (1.6%)
Not first major episode	240(64.7%)	273 (71.1%)	513 (67.9%)
Missing	108 (29.1%)	82 (21.3%)	190 (25.2%)
4+ previous episodes	162 (43.7%)	227 (59.1%)	389 (51.5%)
Missing	133 (35.8%)	113 (29.4%)	246 (32.6%)
Age at first major depressive episode			
Mean (SD)	24.89 (11.56)	25.47 (12.9)	25.2 (12-28)
Median (min, max)	21 (6, 72)	23 (0-79)	22(0-79)
IQR (25%, 75%	16, 32	16, 31	16,31
Missing	7 (0.9%)	9(1.2%)	16 (2.1%)
Medication			
Depression medication in last 3 months	250 (67.3%)	269 (70%)	519 (68.7%)
Missing	-	-	-
Analgesic medication in last 3 months	114 (30.7%)	245 (63.8%)	359 (47.5%)
Missing	4 (1%)	2 (0.52 %)	6 (0.8%)
EQ-5D Pain			
No pain	371 (49.1%)	0	371 (49.1%)
Moderate pain or discomfort	0	298 (39.5%)	298 (39.5%)
Extreme pain or discomfort	0	86 (11.4%)	86 (11.4%)
EQ-5D Anxiety/Depression			
Not anxious/depressed	13 (3.5%)	8 (2.1%)	21 (2.8%)
Moderately anxious/depressed	282 (76%)	272 (70.8%)	554 (73.4%)
Extremely anxious/depressed	74 (19%)	104 (27%)	178 (23.6%)
Missing	2 (0.5%)	0	2 (0.3%)
EQ5D Mobility			
No problems walking about	361 (31%)	234 (31%)	595 (79%)
Some problems walking about	9(1.2%)	148(19.7%)	157(20.8%
Confined to bed	0(0%)	1(0.1%)	1(0.1%)
Missing	-	-	-
EQ5D Self-Care			
No problems with self-care	358 (47.5%)	314 (41.7%)	672 (89.2%)
Some problems with self-care	11(1.5%)	68 (9%)	79 (10.5%)
Unable to wash or dress	1(0.1%)	1 (0.1%)	2 (0.3%)
Missing	1(0.1%)	1(0.1%)	2(0.3%)
EQ5D Usual activities			
No problems performing usual activities	251(33.2%)	111(14.7%)	362(48.1%)
Some problems performing usual activities	115(15.3%)	247(32.8%)	115(48.1%)
Unable to perform usual activities	5 (0.7%)	24 (3.2%)	29(3.9%)
Missing	2(0.3%)	0(0%)	2(0.3%)
PHQ-9			
Mean (SD)	14.97 (5.15)	17.0 (5.23)	16.0 (5.29)
Median (min, max)	15 (3, 27)	17 (3, 27)	16 (3-27)
IQR (25%, 27%)	11, 19	13, 21	12, 20
Missing	-	-	1 (0.1%)
BDI-II			
Mean (SD)	31.32 (8.29)	33.6 (8.9)	32.5 (8.72)
BDI-II Group			
Moderate (20-28)	155 (41.8%)	129 (33.6%)	284 (37.6%)
Severe (29-63)	216 (52.8%)	255 (66.4%)	471 (62.4%)
Trial arm allocation			
Acupuncture	156 (20.7%)	146 (19.3%)	302(40%

For peer review only

Counselling	151 (20%)	151 (20%)	302(40%)
Usual Care	64 (8.5%)	87 (11.5%)	151(20%)
Missing	-	-	-
Expectation of treatment allocated			
Very Ineffective	13 (3.5%)	21 (9.6%)	34 (4.5%)
Fairly Ineffective	39 (10.5%)	57 (14.8%)	96 (12.8%)
Can't decide	159 (42.9%)	178 (46.4%)	337(44.9%)
Fairly effective	122 (32.9%)	87 (22.7)	209 (27.8%)
Very effective	38 (10.2%)	37 (9.6%)	75(10.0%)
Missing	0	4 (0.5%)	4 (0.5%)
Treatment Preference			
Acupuncture	196 (52.8%)	234 (60.9%)	430 (57.5%)
Counselling	94 (25.3%)	70 (18.2%)	164(21.9%)
Usual Care	3 (0.8%)	7 (1.8%)	10 (1.3%)
No preference	76 (20.5%)	68 (17.7%)	144 (19.3%)
Missing	2 (0.5%)	5 (1.3%)	7 (0.9%)

Objective 2: Do patients reporting pain at baseline have different outcomes over time and in response to treatment?

Does baseline pain impact on depression at 3 months?

A linear regression model found that the presence of pain at baseline predicted a poorer outcome of depression treatment at 3 months, whilst controlling for baseline depression scores (β=1.717, α=4.483, t= 3.685, df2, p<0.001) and accounted for approximately 25% of the variance in the model (R sqd = 0.248, df2, p<0.001).

Does baseline pain impact on depression at 3 months if other demographic predictors are taken into account?

A systematic univariate analyses of demographic variables, the BDI-II depression items and the five EQ-5D items, whilst controlling for PHQ-9 scores at baseline identified 11 possible predictors (p<0.1) of PHQ-9 depression at 3 months (Supplement 1,Web only file). In a second stage regression model including these predictors simultaneously, three remained significant predictors of depression (increased problems with mobility, a greater loss of energy, and sex where males had worse depression at 3 months) in addition to the presence of moderate to extreme pain and baseline PHQ-9 depression (Supplement2, Web only file). The variables identified were then included in a final linear regression model (p<0.05). See table 2.

Table 2. Regression model predicting PHQ-9 depression at 3 months. (p <0.05)

Predictor variable	β	Std Error	95% Confidence Interval	Sig p
Constant	4.605	1.457	1.744 to 7.465	.002
Pain group/no pain group allocation based on EQ-5D at baseline	-1.011	0.507	-2.007 to -0.15	.047
EQ-5D mobility at baseline	1.760	0.635	.513 to 3.007	.006
BDI-II q15Loss of energy at baseline	0.922	0.355	.224 to 1.620	.010
Sex- being male	1.036	0.512	-2.041 to -0.32	.043
PHQ-9 score at baseline	0.480	0.047	.387 to 573	.000

Does baseline pain moderate the response to treatment at 3 months?

Figure 1 presents PHQ-9 depression scores by pain group and by trial arm at baseline and all follow up time points. Using the average across all treatment groups, participants in the pain group showed less reduction in depression scores at three months compared to baseline (F, 1,603 = 4.848, p<0.028) mean 16.7 to 12.06 at 3 months) than the no pain comparator group (baseline mean 14.06 to 9.1 at 3 months), a difference that was significant in an ANCOVA model controlling for baseline PHQ-9 (mean difference = 1.16, 95%CI = 0.12 to 2.2). Controlling for baseline depression an ANCOVA model including a pain group by treatment interaction term showed that within the pain group, participants showed a larger reduction in depression with acupuncture at three months (mean reduction in PHQ-9 from baseline (6.0, 95%CI = 5.07 to 7.11), with smaller reductions associated with counselling (4.3, 95%CI = 3.3 to 5.4) and usual care (2.7, 95%CI = 1.50 to 4.06). In comparison, no notable differences were seen between treatment arms within the no pain comparator group.

In an ANCOVA model (controlling for baseline measures of depression, EQ5D mobility, BDI-II loss of energy and sex) of the pain group alone, participants who received acupuncture reported significantly more depression free days between baseline to three months follow up than those receiving usual care alone (mean difference 15.2, 95%CI= 3.12 to 15.05). The differences between counselling and acupuncture, and

For peer review only

between counselling and usual care were not significant. The no pain comparator group also showed a trend in favour of acupuncture. See Table3.

Table 3. Summary of depression free days at three months from baseline by pain group and trial arm.			
	Depression free days		
	No pain	Moderate –Extreme pain	Total
**Treatment received**	Mean (Sd)	Mean (Sd)	Mean (Sd)
**Acupuncture**	37.3 (26.4)	31.3 (23.8)	31.7 (25.3)
**Counselling**	33.0 (22.2)	20.7 (21.9)	26.9 (22.9)
**Usual Care**	32.0 (23.5)	16.1(22.2)	22.6 (23.5)

Does bodily pain change over time as a response to treatment for depression?

Using the SF-36 bodily pain score at 3 months follow up as the endpoint and controlling for baseline SF-36 bodily pain and baseline PHQ-9 depression scores, results of ANCOVA show that the pain group continued to experience significantly worse pain after treatment for depression compared to the no pain comparator group (mean difference =14.57, 95% CI = 9.73 to 19.40). There was also a significant interaction between pain group and treatment arm ($F (2,1) = 3.3$, $p=0.036$ where pain group patients who received acupuncture for depression experienced a greater reduction in SF-36 bodily pain between baseline to three months follow up (mean reduction = 11.2, 95% CI= 7.1 to 15.2), than those who received counselling (mean reduction = 7.6, 95% CI = 3.6 to 11.6) or usual care (mean reduction =7.2, 95% CI= 2.3 to 12.1).

What are the long term effects of treatment for depression and pain?

Figure 1 shows that after the initial reduction in depression from baseline to 3 months, depression scores tended to remain relatively stable during the 6 to 12 month follow up period in both the pain group and no pain comparator group. With regards to pain, a noticeable reduction in pain at 3 months which persisted through to the 12 month follow up point (Figure 2), the median of pain group after 12 months (median = 41, IQ1=31, IQ3 = 62) remained below the trial baseline median of 52 on the SF-36 bodily pain scale

Adverse events

Treatment response may also be moderated by adverse events; 32% of participants in the pain group reported some form of adverse event between baseline and 12 months compared to 15% of the no pain comparator group, and were twice as likely to report an adverse event (OR = 2.05, 95%CI = 1.467 to 2.877).

DISCUSSION

Summary of results

Participants with moderate to severe pain at baseline had worse outcomes for depression than the no pain comparator group in all three treatment arms after controlling for baseline depression. The results of this sub-study support the main results of the ACUDep trial[15] by showing that at 3 months, both acupuncture and counselling interventions were effective for depression compared to usual care alone irrespective of the presence of comorbid pain. The results also extend the findings of the ACUDep trial[15] by showing that both acupuncture and counselling remain effective treatment options for patients with depression who also have comorbid pain. In addition, participants in the pain group had greater reductions in both depression symptoms with acupuncture from baseline to 3 months than those who received counselling or usual care. All treatment options were effective in reducing pain between baseline and 3 months follow up after controlling for baseline pain, however, acupuncture delivered a greater degree of pain relief than counselling or usual care in the short to medium term.

Strengths and Limitations

This study targets the under researched area of how pain may impact on the outcome of different treatments for depression. The research questions were clearly defined to establish the prevalence of pain in a depressed population, to describe the demographic profile of depressed people in pain compared to those who are depressed and pain free, to determine the impact of pain on the treatment outcomes, and in turn determine the effect of treatment for depression on the level of pain reported in the months subsequent to receiving treatment for depression. As a sub study of a larger pragmatic trial, the findings are important for three reasons; firstly the emphasis on external validity with findings that are generalizable to primary care patients with depression across the UK. Secondly, the method of randomisation to treatment allocation is a feature that was maintained through the sub-grouping into a 'pain group' and 'no pain group' to provide control for

For peer review only

temporal effects and random factors that might influence the outcome of treatment. Finally, the standardised treatment protocols allowed for treatment to be tailored to individual patient's needs regarding pain and depression whilst maintaining high standards of care, qualifications and practice experience. In terms of limitations, this study was a sub study of a larger trial which was not powered to detect differences between the subgroups of moderate to extreme pain and no pain. The use of the BDI-II allowed for physical symptoms of depression to act as predictors to treatment outcome at three months follow up, however this outcome was not measured again at 3 months. Therefore, changes to factors such as sleeplessness, energy loss from within the BDI-II could not be assessed; however, these attributes will be explored qualitatively in a follow up paper. A further limitation is that the actual medication prescribed to participants and the frequency of use was not recorded at baseline or as follow up data, therefore the impact of the treatment on the types of anti-depressant and analgesic medication used or the frequency of use could not be established.

Relationship to literature

Incidence of comorbidity

In terms of comorbidity, the estimated prevalence of moderate to extreme pain within our study population of depressed patients is 51%, which is comparable with the 50-66% in previous literature. [1,2,6,24] This is a consequence of the ACUDep trial[15] targeting primary care patients with moderate to severe depression on the BDI-II. The doubling of adverse events reported by patients in moderate to extreme pain may be accounted for by attendance for hospital investigations and overnight admissions in relation to existing illnesses. However, patients with comorbid pain and depression tend to exhibit a cognitive bias specific to negative aspects of health[25] and are more likely to report less favourable outcomes of treatment.[3] Therefore, it is possible that treatment related minor adverse events are more likely to be reported by patients who suffer from chronic pain.

Predictors of depression

Evidence from the English longitudinal study of ageing[26] identified pain and mobility disability at baseline as predictors of comorbid pain and depression. Together these factors are known to adversely affect the outcomes of treatment for depression.[7,8,27] Consistent with previous research, the majority of painful complaints within the study sample were of musculoskeletal origin and accompanied by poor mobility and a loss of energy. Pain due to osteoarthritis is known to determine subsequent depressed mood through its effect on fatigue and disability.[28]

Reduction in pain

The reductions in pain from baseline to 3 months are within the lower range of 5-30 points for a minimally clinically important difference[18,29], however, the majority of patients with pain remained below the trial baseline median of 52 on the SF-36 bodily pain scale at 3 months That patients reported reduced pain following acupuncture, is not surprising; 32% of patients had chronic muscular skeletal pain, for which there is a growing body of evidence supporting the efficacy of acupuncture.[10] With acupuncture being a holistic therapy, it is likely that acupuncturists incorporated treatment for pain alongside treatment for the symptoms of depression. Patients who received counselling also reported a more gradual reduction in pain over the 12 month follow up period. This finding is consistent with a Cochrane Review[30] which reports that psychological therapies, primarily CBT , can help people with people with chronic pain, reduce negative mood (depression and anxiety), disability, and to a lesser degree reduce pain over a six month period.

Implications for clinical practice and future research

Frequently patients with comorbid pain and depression will attribute their complaint to one or other of the conditions and seek help accordingly.[31] Patients presenting with pain or sleep disturbances are at risk of having a diagnosis of depression missed.[27] For treatment success, both pain and depression should be recognized and treated from the outset. The evidence emerging for the current study is that both acupuncture and counselling appear to have the potential to reduce symptoms associated with pain and depression concurrently, potentially affording patients' relief from symptoms of depression and reduced intensity of pain in both the short and longer term. This study provides a platform from which to develop a larger investigation specifically designed with sufficient power to determine the impact of acupuncture or counselling when compared to other treatments for depression in patients with comorbid pain and depression.

CONCLUSION

For peer review only - http://bmjopen.bmj.com/site/about/guidelines.xhtml

Patients who had moderate to extreme pain comorbid with depression at baseline recovered less well when compared to those who were pain free. Over three months, larger reductions in depression and pain scores were found in those who received acupuncture compared to those receiving counselling, and in turn these were greater than those receiving usual care. For those in pain at baseline, both acupuncture and counselling delivered a clinically meaningful reduction in bodily pain over 12 months.

Figure legends:

Figure 1. PHQ-9 depression scores by pain/no pain groups and trial arm at baseline and follow up time points.

Figure 2. A summary of the SF-36 bodily pain scores by pain /no pain group and trial arm at baseline and follow up time points.

Acknowledgements:
We would like to acknowledge the contribution of the patients, counsellors, acupuncturists and general medical practitioners; the ACUDep trial management team and members of MIND York.

Funding:
This article presents independent research funded by the National Institute for Health Research (NIHR) under its Programme Grants for Applied Research Programme (RP-PG-0707-10186). The views expressed are those of the author(s) and not necessarily those of the National Health Service, the NIHR or the Department of Health. The funders, and the University of York in its role as sponsor for the study, had no role in study design, data collection and analysis, decision to publish, or preparation of the manuscript.

Competing interests:
There are no competing interests.

Ethics approval:
Ethical approval was obtained within the ACUDEP trial (ISRCTN63787732) (REF) from the York NHS Research Ethics Committee (ref:09/H1311/75).

Contributors:
Ann Hopton conducted the analysis, interpreted the data, drafted and revised the article.
Hugh MacPherson revised critically important intellectual content and gave final approval for publication.
Ada Keding provided substantial contribution to the conception and design and final approval for publication secondary analysis of the data and final approval for publication.
Stephen Morley provided substantial contribution to the conception and design, and final approval for publication.

REFERENCES

1 Katona C, Peveler R, Dowrick C, *et al.* Pain symptoms in depression: definition and clinical significance. *Clinical Medicine* 2005;**5**:390–5. doi:10.7861/clinmedicine.5-4-390

2 Arnow BA. Comorbid Depression, Chronic Pain, and Disability in Primary Care. *Psychosomatic Medicine* 2006;**68**:262–8. doi:10.1097/01.psy.0000204851.15499.fc

3 Linton SJ, Nicholas MK, MacDonald S, *et al.* The role of depression and catastrophizing in musculoskeletal pain. *European Journal of Pain* 2011;**15**:416–22.

For peer review only

4 CG91: Depression in adults with a chronic physical health problem: Treatment and management.

5 Green A, Crawford A, Lewis G, *et al.* Are baseline physical symptoms characteristic of severe depression or a poor prognostic factor? Secondary analysis of the GenPod trial. *Journal of Psychosomatic Research* 2013;**74**:546. doi:10.1016/j.jpsychores.2013.03.038

6 Bair MJ, Robinson RL, Eckert GJ, *et al.* Impact of pain on depression treatment response in primary care. *PsychosomMed* 2004;**66(1)**:17–22.

7 Kroenke K, Shen J, Oxman TE, *et al.* Impact of pain on the outcomes of depression treatment: Results from the RESPECT trial. *Pain* 2008;**134**:209–15. doi:10.1016/j.pain.2007.09.021

8 DeVeaugh-Geiss AM, West SL, Miller WC, *et al.* The Adverse Effects of Comorbid Pain on Depression Outcomes in Primary Care Patients: Results from the Artist Trial. *Pain Medicine* 2010;**11**:732–41. doi:10.1111/j.1526-4637.2010.00830.x

9 Bergbom S, Boersma K, Overmeer T, *et al.* Relationship Among Pain Catastrophizing, Depressed Mood, and Outcomes Across Physical Therapy Treatments. *Physical Therapy* 2011;**91**:754–64. doi:10.2522/ptj.20100136

10 Vickers AJ. Acupuncture for Chronic Pain: Individual Patient Data Meta-analysis. *Archives of Internal Medicine* 2012;**172**:1444. doi:10.1001/archinternmed.2012.3654

11 Hopton AK, Curnoe S, Kanaan M, *et al.* Acupuncture in practice: mapping the providers, the patients and the settings in a national cross-sectional survey. *BMJ Open* 2012;**2**.http://bmjopen.bmj.com/content/2/1/e000456.abstract

12 Thomas KJ, Nicholl JP, Coleman P. Use and expenditure on complementary medicine in England: a population based survey. *Complement TherMed* 2001;**9**:2–11.

13 Mellor-Clark J, Simms-Ellis R, Burton M. National survey of counsellors working in primary care: evidence for growing professionalisation? *Occas Pap R Coll Gen Pract* 2001;**79**:vi–7.

14 Bower P, Knowles S, Coventry PA, *et al.* Counselling for mental health and psychosocial problems in primary care. *CochraneDatabaseSystRev* 2011;:CD001025.

15 MacPherson H, Richmond S, Bland M, *et al.* Acupuncture and Counselling for Depression in Primary Care: A Randomised Controlled Trial. *PLoS Medicine* 2013;**10**:e1001518. doi:10.1371/journal.pmed.1001518

16 MacPherson H, Richmond S, Bland MJ, *et al.* Acupuncture, Counseling, and Usual care for Depression (ACUDep): study protocol for a randomized controlled trial. *Trials* 2012;**13**:209.

17 Beck A, Ward C, Mendelson M, *et al.* An inventory for measuring depression. *ArchGenPsychiatry* 1961;**4**:561–71.

18 Garratt A, Ruta D, Abdalla M, *et al.* The SF36 health survey questionnaire: an outcome measure suitable for routine use within the NHS? *BMJ* 1993;**306**:1440–4.

19 MacPherson H, Elliot B, Hopton A, *et al.* Acupuncture for Depression: Patterns of Diagnosis and Treatment within a Randomised Controlled Trial. *Evidence-Based Complementary and Alternative Medicine* 2013;**2013**:1–9. doi:10.1155/2013/286048

20 Roth A, Hill A, Pilling S. The competences required to deliver effective Humanistic Psychological Therapies. *University College London, Centre for Outcomes Research and Effectiveness*;:2009.

21 Kroenke K, Spitzer RL, Williams JB. The PHQ-9: validity of a brief depression severity measure. *J Gen Intern Med* 2001;**16**:606–13.

For peer review only

22 Mischoulon D, Brill CD, Ameral VE, *et al.* A pilot study of acupuncture monotherapy in patients with major depressive disorder. *Journal of Affective Disorders* 2012;**141**:469–73. doi:10.1016/j.jad.2012.03.023

23 Vannoy SD, Arean P, Unutzer J. Advantages of using estimated depression-free days for evaluating treatment efficacy. *Psychiatr Serv* 2010;**61**:160–3.

24 Kroenke K, Wu J, Bair MJ, *et al.* Reciprocal Relationship Between Pain and Depression: A 12-Month Longitudinal Analysis in Primary Care. *The Journal of Pain* 2011;**12**:964–73. doi:10.1016/j.jpain.2011.03.003

25 Rusu AC, Pincus T, Morley S. Depressed pain patients differ from other depressed groups: Examination of cognitive content in a sentence completion task. *PAIN* 2012;**153**:1898–904. doi:10.1016/j.pain.2012.05.034

26 Chou K-L. Combined effect of vision and hearing impairment on depression in older adults: Evidence from the English Longitudinal Study of Ageing. *Journal of Affective Disorders* 2008;**106**:191–6. doi:10.1016/j.jad.2007.05.028

27 Ohayon MM, Schatzberg AF. Chronic pain and major depressive disorder in the general population. *Journal of Psychiatric Research* 2010;**44**:454–61. doi:10.1016/j.jpsychires.2009.10.013

28 Hawker GA, Gignac MAM, Badley E, *et al.* A longitudinal study to explain the pain-depression link in older adults with osteoarthritis. *Arthritis Care & Research* 2011;**63**:1382–90. doi:10.1002/acr.20298

29 White DK, Wilson JC, Keysor JJ. Measures of adult general functional status: SF-36 Physical Functioning Subscale (PF-10), Health Assessment Questionnaire (HAQ), Modified Health Assessment Questionnaire (MHAQ), Katz Index of Independence in Activities of Daily Living, Functional Independ. *Arthritis Care & Research* 2011;**63**:S297–S307. doi:10.1002/acr.20638

30 Eccleston C, Williams AC de C, Morley S. Psychological therapies for the management of chronic pain (excluding headache) in adults. In: The Cochrane Collaboration, Eccleston C, eds. *Cochrane Database of Systematic Reviews*. Chichester, UK: : John Wiley & Sons, Ltd 2009. http://doi.wiley.com/10.1002/14651858.CD007407.pub2 (accessed 5 Dec2013).

31 Robinson MJ, Edwards SE, Iyengar S, *et al.* Depression and pain. *Front Biosci (Landmark Ed)* 2009;**14**:5031–51.

For peer review only

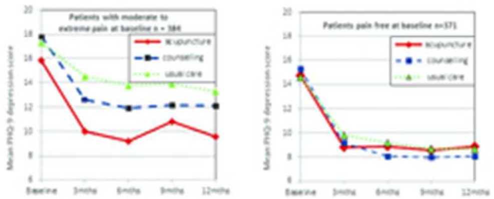

Figure 1. PHQ-9 depression scores by pain/no pain groups and trial arm at baseline and follow up time points.
15x11mm (600 x 600 DPI)

review only

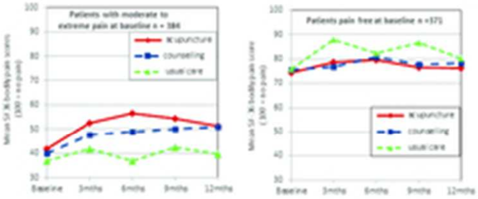

Figure 2. A summary of the SF-36 bodily pain scores by pain /no pain group and trial arm at baseline and follow up time points.
15x11mm (600 x 600 DPI)

review only

Supplement 1. Univariate analyses of potential predictors of PHQ-9 depression at 3 months, controlling for baseline PHQ-9

**Demographic Items**	**F(df), p**
Age	F(1,65) = 1.057, p = 0.363
Sex*	F(1,1) = 1.249, p = 0.040
Age left education	F(1,24) = 1.258, p = 0.186
Employment*	F(1,7) = 2.072, p = 0.045
Painful health or medical condition*	F(1,2) = 6.389, p = 0.002
N Episodes of depression*	F(1,2) = 4.129, p = 0.017
Age at onset of depression	F(1,60) = 1.073, p = 0.337
Medication- Analgesics*	F(1,2) = 7.996, p = 0.001
Medication - Antidepressants	F(1,2) = 2.554, p = 0.111
EQ5D Anxiety	F(1,3) = 0.097, p = 0.406
Expectations of allocated treatment	F(1,4) = 1.035, p = 0.389
Preference for treatment	F(1,4) = 1.193, p = 0.313
**BDI-II Items**	
1. Sadness	F(1,3) = 0.185, p = 0.907
2.Pessimism	F(1,3) = 0.833, p = 0.476
3.Past Failure	F(1,3) = 1.717, p = 0.162
4.Loss of Pleasure	F(1,3) = 0.944, p = 0.395
5.Guilty feelings	F(1,3) = 1.820, p = 0.142
6.Punishment feelings	F(1,3) = 0.784, p = 0.503
7.Self-Dislike	F(1,3) = 0.856, p = 0.503
8.Self Criticalness	F(1,3) = 0.054, p = 0.983
9.Suicidal thoughts or Wishes*	F(1,3) = 2.655, p = 0.048
10.Crying	F(1,3) = 0.108, p = 0.955
11.Agitation	F(1,3) = 0.756, p = 0.529
12.Loss of Interest	F(1,3) = 0.483, p = 0.694
13. Indecisiveness	F(1,3) = 1.820, p = 0.142
14.Worthlessness	F(1,3) = 1.834, p = 0.140
15.Loss of Energy*	F(1,3) = 4.243, p = 0.006
16.Changes in Sleeping Pattern	F(1,3) = 3.129, p = 0.025
17.Irritability	F(1,3) = 0.205, p = 0.893
18. Changes in appetite	F(1, 3) = 0.921, p = 0.893
19. Concentration Difficulty	F(1,3) = 1.307, p = 0.271
20. Tiredness or fatigue*	F(1,3) = 2.485, p = 0.060
21. Loss of Interest in Sex	F(1,3) = 1.963, p = 0.118
**Other EQ5D Variables at baseline**	
Mobility*	F(1,2) = 13.673, p = 0.001
Self-Care *	F(1,2) = 5.369, p = 0.001
Activity*	F(1,2) = 8.850, p = 0.001
Anxiety	F(1,2) = 0.972, p = 0.406

NB *denotes p<0.10

Supplement 2. Linear regression of possible variables predicting PHQ-9 depression at 3 months. (p <0.1)				
**Predictor variable**	**β**	**Std Error**	**95% Confidence Interval**	**Sig p**
Constant	5.170	1.336	2.54 to 7.79	0.000
Pain group/no pain group allocation based on EQ-5D*	-0.777	0.463	-1.68 to 0.13	0.09
Baseline EQ5D Self-Care	1.063	0.600	-0.11 to 2.24	0.32
Baseline prescribed analgesics	-0.002	0.002	.27 to - 0.007	0.27
Baseline EQ-5D Performing usual activity	0.272	0.401	-0.51 to 1.06	0.49
Type of health problem	-0.111	0.154	-0.41 to 0.19	0.47
BDI-II Question 15 "Loss of energy"*	0.644	0.355	-0.55 to 1.13	0.07
Previous episodes of depression	0.044	0.115	-0.22 to 0.23	0.97
Baseline BDI-II Question 16 "Changes in sleeping pattern"	0.052	0.234	-0.40 to 0.51	0.82
Sex- being male*	-0.840	0.436	-1.69 to -0.15	0.05
Employment	0.142	0.099	-0.05 to 0.33	0.15
Baseline BDI Question 20 "Tiredness or fatigue"	0.013	0.326	-0.628 to 0.652	0.96
EQ-5D mobility at baseline*	1.760	0.635	.51 to 3.00	0.006
Baseline PHQ-9 score*	0.480	0.047	.38 to 573	0.000

For peer review only

BMJ Open

ACUPUNCTURE, COUNSELLING OR USUAL CARE FOR DEPRESSION AND COMORBID PAIN: SECONDARY ANALYSIS OF A RANDOMISED CONTROLLED TRIAL

Journal:	*BMJ Open*
Manuscript ID:	bmjopen-2014-004964.R1
Article Type:	Research
Date Submitted by the Author:	11-Apr-2014
Complete List of Authors:	Hopton, Ann; University of York, Health Sciences<br>MacPherson, Hugh; University of York, Health Sciences<br>Keding, Ada; University of York, Health Sciences<br>Morley, Stephen; University of York, Health Sciences
**Primary Subject Heading**:	Complementary medicine
Secondary Subject Heading:	Mental health, General practice / Family practice, Evidence based practice
Keywords:	Depression, Comorbid pain, Acupuncture, Counselling, Usual Care

SCHOLARONE™
Manuscripts

For peer review only

ACUPUNCTURE, COUNSELLING OR USUAL CARE FOR DEPRESSION AND COMORBID PAIN: SECONDARY ANALYSIS OF A RANDOMISED CONTROLLED TRIAL

A. Hopton[1], H. MacPherson[1], A. Keding [1], S. Morley[2]

[1] Department of Health Sciences, University of York, UK

[2]Leeds Institute of Health Sciences - University of Leeds

Address for correspondence:

Ann Hopton
Research Fellow
Department of Health Sciences
University of York
Heslington
York YO10 5DD
United Kingdom
Tel: 01904 321384
E-mail: ann.hopton@york.ac.uk

Keywords: Depression, comorbid pain, acupuncture, counselling, usual care
Words 4,082.

For peer review only

ABSTRACT

Background
Depression with comorbid pain is associated with a poor response to various treatments. The objective in this secondary analysis is to determine whether patients reporting pain have different depression and pain outcomes over time in response to acupuncture, counselling or usual care.

Methods
Self-reported ratings of depression and pain from 755 patients in a pragmatic randomised controlled trial of acupuncture (302) or counselling (302) compared to usual care alone (151) are described and analysed using a series of regression models and ANCOVA. Patients reported outcomes of PHQ-9 for depression, SF36 bodily pain and EQ-5D, all at baseline, 3, 6, 9, and 12 months.

Results
At baseline, 755 patients reported EQ-5D pain categories; 384 (50.9%) reported moderate to extreme pain. Controlling for baseline depression, a linear regression model showed that the presence of pain at baseline was associated with poorer depression outcomes at 3 months mean difference = -1.16, (95%CI = 0.12 to 2.2) Participants with moderate to extreme pain at baseline did better at 3 months if they received acupuncture (mean reduction in PHQ-9 from baseline = 6.0, 95%CI = 5.0 to 7.1 and a mean reduction in SF-36 bodily pain = 11.2, (95% CI= 7.1 to 15.2) compared to improvements for those who received counselling (4.3, 95%CI = 3.3 to 5.4; 7.6, 95% CI = 3.6 to 11.6) or usual care (2.7, 95%CI = 1.50 to 4.0: 7.2, 95% CI= 2.3 to 12.1). In comparison, no notable differences were seen between treatment arms within the no pain comparator group.

Conclusion
Patients with depression and pain at baseline recovered less well from treatment over three months than those with depression and no pain. Reductions in both depression and pain were most marked in the acupuncture group, followed by the counselling group, and then the usual care group.

(299 words)

Strengths:

- This study targets the under researched area of how pain may impact on the outcome of different treatments for depression.
- The research questions were clearly defined to establish the prevalence of pain in a depressed population, to describe the demographic profile of depressed people in pain compared to those who are depressed and pain free, to determine the impact of pain on the treatment outcomes, and in turn determine the effect of treatment for depression on the level of pain reported in the months subsequent to receiving treatment for depression.
- As a sub study of a larger pragmatic trial, the findings are important for three reasons; firstly the emphasis on external validity with findings that are generalizable to primary care patients with depression across the UK. The method of randomisation to treatment allocation is a feature that was maintained through the sub-grouping into a 'pain group' and 'no pain group' to provide control for temporal effects and random factors that might influence the outcome of treatment.

Limitations:

- This study was a sub study of a larger trial which was not powered to detect differences between the subgroups of moderate to extreme pain and no pain.
- The use of the BDI-II outcome was not measured at 3 months.
- Changes to factors such as sleeplessness, energy loss from within the BDI-II could not be assessed at the primary outcome point.
- The name of the medication prescribed to participants and the frequency of use was not recorded at baseline or as follow up data, therefore the impact of the treatment on the types of anti-depressant and analgesic medication used or the frequency of use could not be established.

For peer review only

INTRODUCTION

Around 50%-66% of patients who are depressed also report pain.[1,2] Depression accentuates existing pain problems[3] adding to the emotional distress and poor sleep irrespective of whether the pain had a known cause[4]. Increased pain symptoms may also represent a greater severity of depression rather than a poor prognostic factor.[5] In terms of treatment, comorbid pain and depression are present in two thirds of depressed primary care patients who commence antidepressant therapy.[6] Pain is known to interfere with the routine activities in 42% of primary care patients prior to starting pharmacological treatment for depression[7] and has a strong negative impact on achieving remission.[8] Too frequently there is a focus on the treatment of either pain or depression. The use of complementary therapies alongside conventional treatments for depression is widespread; this uptake may be attributed to the perceptions of ineffectiveness to patients, the distress of continued symptoms and the unwanted side effects of medication [9].Treatment outcomes may be enhanced by taking an holistic approach, and delivering treatment options that address both pain and depression together.[10]

A meta-analysis of 18,000 participants data from 25 high quality trials has shown that acupuncture is an effective treatment option for several chronic pain conditions.[11] Acupuncture is also regularly provided by acupuncturists to treat depression[12] although not routinely available as a treatment option for depression within the UK's National Health Service.[13] Counselling for depression is widely available in primary care practices[14] however there is limited evidence for counselling compared to usual care as a treatment for patients with depression and a chronic physical health problem.[4,15] A recent randomised controlled trial of acupuncture or counselling provided to patients with depression found that both interventions significantly reduced depression at three months when compared to usual care.[15] Pain was a secondary outcome in this trial, but has not yet been reported, and it will be of interest to compare the treatment outcomes of patients who had comorbid pain and depression with those who had depression alone.

The primary aim of this study is to find out whether people with depression who are also in pain have better or worse depression outcomes than those without pain. In terms of objectives, we document the prevalence of pain in a depressed population, the demographic profile of patients in pain and the relationship between pain and depression. We then determine depression and pain outcomes following treatment with acupuncture or counselling when compared to usual care alone.

METHODS

Design

This research is a sub-study nested within a three-arm randomised controlled trial of acupuncture or counselling as adjuncts to usual care compared to usual care alone.[15] Full details of the ACUDep trial protocol are reported elsewhere.[15,16] Whilst maintaining the integrity of the 2:2:1 randomisation allocation of the ACUDep trial[16], for the purpose of this sub-study participants were divided into two groups according to their response to the EQ-5D pain statements at baseline: I have no pain or discomfort; I have moderate pain or discomfort; I have extreme pain or discomfort. People who reported either moderate or extreme pain were considered together as the "pain group", the remainder were assigned as a "no pain comparator group". A summary of the sub-group allocations and other demographic variables are presented in Table 1.

Participants

Recruited to the main ACUDep trial[15] were 755 patients aged 18 or over and diagnosed with depression or who had consulted for depression within the previous 30 months with a baseline score of 20 or above on the Beck Depression Inventory-II, which this scale classes as "moderate" to "severe" depression.[17] Patients were recruited from general practices in rural and urban areas of Yorkshire, County Durham and Northumberland. Bodily pain scores on the SF-36[18] and any chronic physical conditions or pre-existing illnesses were reported by patients at baseline. Patients' details were recorded by the York Trials Unit and randomised by computer-generated block randomisation, with block sizes of 5 and 10 by an investigator with no clinical attachment to the trial. An unequal allocation ratio of 2:2:1 to acupuncture, counselling and usual care provided groups of 302, 302 and 151 respectively.

Interventions

For peer review only

Within the main ACUDep trial[15] participants allocated to the acupuncture and counselling groups received the offer of up to 12 sessions on a weekly basis. The acupuncture intervention was performed according to a treatment protocol developed and agreed by acupuncture practitioners, which allowed for individualised treatment within a standardised theory-driven framework. The acupuncturists were members of the British Acupuncture Council with a minimum post qualification experience of three years, specific details of the intervention are published elsewhere[19] The treatment protocol for the counselling intervention was based on competences independently developed for Skills for Health within National Occupational Standards for Psychological Therapies.[20] The counsellors were members of the British Association for Counselling and Psychotherapy with a minimum of 400 hours post qualification experience and used a non-directive approach to help clients express feelings, clarify thoughts, and reframe difficulties. Usual care available to all participants throughout the trial included prescribed pharmaceutical and other interventions provided by NHS primary or secondary mental health services.

Outcome measures

The pain and depression outcome measures used for this sub-study were collected as part of the ACUDep trial[15]. These included the primary depression score as measured by the Patient Health Questionnaire (PHQ-9)[21], which scores each of the 9 DSM-IV criteria for depression as “0” (not at all) to “3” (nearly every day). The primary endpoint was at 3 months, with further follow up at 6, 9 and 12 months. The patients’ experience of the effect of the treatment received can also be expressed as depression free days[22,23] which is an approximate summary measure derived from the PHQ-9 cut off scores averaged over the period between measurements[15]. Patient reported pain measures were collected at baseline and at 3, 6, 9, and 12 months follow up using the SF-36 Bodily Pain sub-scale, which has a range 0-100, where a score of 100 indicated no pain, and the EQ-5D categories of no pain or moderate pain or severe pain. At baseline, patients also recorded their use of anti-depressants and analgesic medication, and their preferences and expectations of treatment.

Data analysis

Prevalence and demographic profile of patients with pain

Descriptive statistics for EQ-5D pain and SF-36 pain scores were calculated. Based on the EQ-5D response categories, patients were allocated to a ‘no pain’ and ‘moderate or extreme pain’ group, using the median SF-36 cut-off where EQ-5D data were missing. A descriptive analysis of the demographic profile of the pain group and no pain comparator group was conducted: The participants’ gender, average age of the sample population and pain groups population, their age on leaving education, the onset of their depression, the number of episodes they experienced, and the baseline scores of PHQ-9 and BDI-II baseline, SF-36 bodily pain score reported.

Pain and depression at baseline

ANOVA was used to compare the mean baseline PHQ-9 scores between the pain group and no pain comparator group. For the pain group alone, a Kendall’s Tau correlation was used to test the association between the baseline scores of the PHQ-9 and SF-36 bodily pain score. This test was considered the most appropriate given the possibility of tied scores between the PHQ-9 and SF-36 bodily pain.

Baseline pain and depression at three months

A series of regression models was applied to determine the influence of baseline pain in the presence of other demographic variables on the PHQ-9 depression outcome at 3 months, the primary endpoint of the trial. The first model predicted PHQ-9 at 3 months from the baseline EQ-5D Pain Group (‘pain’ or ‘no pain’), controlling for baseline PHQ-9 scores. Additional predictors of PHQ-9 depression at 3 months were identified by individual univariate analyses of demographic variables, the BDI-II depression items and the five EQ-5D items whilst controlling for PHQ-9 scores at baseline. For this scoping exercise, a less conservative level of significance was set at $p<0.1$ in order for potential covariates not to be missed and to maintain consistency with the methodology of the main ACUDep trial analysis. Any variables identified by univariate analysis were then included in a combined linear regression model, and any significant covariates were taken forward to the final model including pain grouping. Controlling for any remaining significant covariates of the final model ($p<0.05$) and baseline PHQ-9 depression scores, ANCOVA was used to test whether baseline pain affected treatment outcomes measured by the PHQ-9 score at three months.

Normality of continuous variables was assessed by inspection of histograms and Q-Q plots. All predictor variables included in the combined models were checked for collinearity in a correlation matrix and through assessment of tolerance and variance inflation factor (VIF) values.

For peer review only

Baseline pain and treatment outcomes (depression and pain)

An interaction term between treatment and pain group in the above ANCOVA model was used to establish whether patients in pain responded differently to the treatments with regards to their PHQ-9 depression at 3 months than patients reporting no pain. The analysis was repeated for the outcome of depression free days at three months, an approximate summary measure derived from PHQ-9 cut-off scores averaged over the period between measurements.[15,23] Descriptive analysis of the depression and pain scores was conducted over the 12 month follow up period.

Adverse events

A comparison of the adverse events reported between baseline and 12 month follow up was conducted using proportions and odds ratio.

Missing data

Results of the main ACUDep analysis revealed an average response rate of 81% for the PHQ-9 at 3 months follow-up, and the primary analysis employed multiple imputation by chained regression using selected demographics and baseline pain and depression outcomes to impute PHQ-9 scores. For the present secondary analysis, imputation was not considered. However, for the initial allocation of patients into 'pain' and 'no pain' sub-groups, the SF-36 bodily pain scale was used where the baseline EQ-5D pain item was not available in order to utilise all available outcome data. Outcomes were assumed to be missing at random, and the analysis of patients in their randomly allocated treatment groups (intention to treat) aimed to control for any random factors that might have influenced the outcome of treatment.

RESULTS

An exploration of pain and depression at baseline

What is the prevalence of pain?

Patients reported pain or discomfort on the EQ-5D questionnaire at baseline; 371 (49.1%) reported no pain or discomfort, 298 (39.5%) reported moderate pain or discomfort and 86 (11.4%) reported extreme pain or discomfort. The distribution of SF-36 bodily pain scores was negatively skewed; 140 (18.5%) patients reported a score of 100, indicating no pain at all, and the sample returned a median pain score of 52 (Interquartile 1= 25, Interquartile 3 = 84).

Allocation to "pain" and "no-pain" sub-groups

For the purpose of this study, those reporting moderate and extreme pain or discomfort on the EQ-5D questionnaire were merged together to form a single 'pain group' (n=384, 51%) with the remainder forming the 'no pain' comparator group (n=371, 49%). Patients who omitted to answer the EQ-5D pain question at baseline (n=7; 1%) were assigned to a group according to their SF-36 bodily pain score; 3 scored above the 3rd interquartile range of the SF-36 bodily pain scale and were assigned to the no pain group, the remaining four patients at baseline scored below the median and were assigned to the pain group. Within the defined pain and no-pain subgroups, trial arm allocations retained the 2:2:1 ratio of the original ACUDep[15] randomisation process.

What is the demographic profile of patients in pain and not in pain?

A summary of the demographic profiles, presented in Table 1, show that the pain and the no-pain groups were comparable for most baseline variables. Notable exceptions were: The pain group members tended to be older than the no pain comparator group (mean of 47 years, vs. 40 years) a difference which remained notable after temporarily removing ten pain group members with outlying ages between of 76-93 years. In terms of health and employment, 56% of the pain group (vs. 9% of the no-pain group) reported a painful health condition or illness that predated the onset of depression, for which 64% (vs. 31%) used analgesic medication regularly, 32% (vs. 9%) were unable to work or retired.

What is the relationship between pain and depression at baseline?

The baseline PHQ-9 depression scores indicate that the pain group reported higher levels of depression at baseline (mean PHQ-9=17.0, SD 5.2) than the no pain comparator group (mean PHQ-9=14.9, SD 5.2). Results of ANOVA confirmed the difference to be highly significant (mean difference 2.02, 95%CI =1.28 to 2.76). For the pain group alone the correlation between the PHQ-9 scores and SF-36 bodily pain scores was weak, but highly significant (Kendall's Tau -0.172, p<0.001).

For peer review only

Table 1. Demographics and variables of interest at baseline			
Characteristic	No Pain N= 371 (49%)	Moderate-extreme pain N= 384 (51%)	Total N=755 (100%)
Age			
Mean (SD)	39.9 (11.58)	46.83 (14.11)	43.5 (13.37)
Median (min, max)	39 (18,75)	46 (18,93)	43 (18,93)
IQR (25%, 75%)	31,48	38,56	33,53
Missing	-	-	-
Sex			
Male	88 (23.7%)	113(29.4%)	201 (26.6%)
Female	283 (76.3%)	271(70.6%)	554(73.4%)
Missing	-	-	-
Age left education			
Mean (SD)	18.3 (3.90)	17.53 (4.61)	18.0 (4.37)
Median (min, max)	17.0 (13, 52)	16 (14, 54)	16 (13-54)
IQR (25%, 75%)	16, 21	16,18	16,19
Missing	-	-	-
Employment			
Full-time education	13 (3.5%)	10 (2.6%)	23 (3.1%)
Working full-time	167 (45%)	114 (29.7%)	281 (37%)
Working part-time	83 (22.4%)	61 (15.9%)	144 (19.5%)
Unable to work	19 (5.1%)	76 (19.8%)	95 (12.9%)
Looking after home	41 (11.1%)	42 (10.9%)	83 (11%)
Retired	14 (3.8%)	51 (13.3%)	65(8.8%)
Other	26 (7%)	22 (5.7%)	48(6.5%)
Missing	8 (2.1 %)	8 (2.1%)	16(2.1%)
Painful health or medical condition			
Current painful health or medical condition	41(12.7%)	299 (77.9%)	346 (45.8%)
Onset before depression	32(8.6%)	216 (56.2%)	248 (32.8%)
Missing	2(0.5%)	4(1%)	6 (0.8%)
Type of health problem			
Musculoskeletal	21(6%)	221(58%)	242(32%)
Other	27(7%)	81 (21%)	108(14%)
No health problem	321(87%)	81 (21%	402 (53%)
SF-36 Bodily pain			
Mean (SD)	76.97(22.53)	39.03(22.5)	57.6 (28.44)
Median (min, max)	77.5 (12, 100)	41 (0, 100)	52 (0, 100)
IQR (25%, 75%)	62, 100	22, 51	32, 84
Missing	3 (0.39%)	1 (1.3%)	4 (0.5%)
Depression			
In last 2 weeks	264 (71.2%)	310 (80.7%)	574 (76.1%)
Missing	2 (0.5%)	10 (2.6%)	12 (1.6%)
Not first major episode	240(64.7%)	273 (71.1%)	513 (67.9%)
Missing	108 (29.1%)	82 (21.3%)	190 (25.2%)
4+ previous episodes	162 (43.7%)	227 (59.1%)	389 (51.5%)
Missing	133 (35.8%)	113 (29.4%)	246 (32.6%)
Age at first major depressive episode			
Mean (SD)	24.89 (11.56)	25.47 (12.9)	25.2 (12-28)
Median (min, max)	21 (6, 72)	23 (0-79)	22(0-79)
IQR (25%, 75%)	16, 32	16, 31	16,31
Missing	7 (0.9%)	9(1.2%)	16 (2.1%)
Medication			
Depression medication in last 3 months	250 (67.3%)	269 (70%)	519 (68.7%)
Missing	-	-	-
Analgesic medication in last 3 months	114 (30.7%)	245 (63.8%)	359 (47.5%)
Missing	4 (1%)	2 (0.52 %)	6 (0.8%)
EQ-5D Pain			
No pain	371 (49.1%)	0	371 (49.1%)
Moderate pain or discomfort	0	298 (77.6%)	298 (39.5%)
Extreme pain or discomfort	0	86 (22.4%)	86 (11.4%)
Missing	3	4	7 (0.9%)

Table 1. Demographics and variables of interest at baseline			
Characteristic	No Pain N= 371 (49%)	Moderate-extreme pain N= 384 (51%)	Total N=755 (100%)
EQ-5D Anxiety/Depression			
Not anxious/depressed	13 (3.5%)	8 (2.1%)	21 (2.8%)
Moderately anxious/depressed	282 (76%)	272 (70.8%)	554 (73.4%)
Extremely anxious/depressed	74 (19%)	104 (27%)	178 (23.6%)
Missing	2 (0.5%)	0	2 (0.3%)
EQ5D Mobility			
No problems walking about	361 (31%)	234 (31%)	595 (79%)
Some problems walking about	9(1.2%)	148(19.7%)	157(20.8%
Confined to bed	0(0%)	1(0.1%)	1(0.1%)
Missing	-	-	-
EQ5D Self-Care			
No problems with self-care	358 (47.5%)	314 (41.7%)	672 (89.2%)
Some problems with self-care	11(1.5%)	68 (9%)	79 (10.5%)
Unable to wash or dress	1(0.1%)	1 (0.1%)	2 (0.3%)
Missing	1(0.1%)	1(0.1%)	2(0.3%)
EQ5D Usual activities			
No problems performing usual activities	251(33.2%)	111(14.7%)	362(48.1%)
Some problems performing usual activities	115(15.3%)	247(32.8%)	115(48.1%)
Unable to perform usual activities	5 (0.7%)	24 (3.2%)	29(3.9%)
Missing	2(0.3%)	0(0%)	2(0.3%)
PHQ-9			
Mean (SD)	14.97 (5.15)	17.0 (5.23)	16.0 (5.29)
Median (min, max)	15 (3, 27)	17 (3, 27)	16 (3-27)
IQR (25%, 75%)	11, 19	13, 21	12, 20
Missing	-	1 (0.2%)	1 (0.1%)
BDI-II			
Mean (SD)	31.32 (8.29)	33.6 (8.9)	32.5 (8.72)
BDI-II Group			
Moderate (20-28)	155 (41.8%)	129 (33.6%)	284 (37.6%)
Severe (29-63)	216 (52.8%)	255 (66.4%)	471 (62.4%)
Trial arm allocation			
Acupuncture	156 (20.7%)	146 (19.3%)	302(40%
Counselling	151 (20%)	151 (20%)	302(40%)
Usual Care	64 (8.5%)	87 (11.5%)	151(20%)
Missing	-	-	-
Expectation of treatment allocated			
Very Ineffective	13 (3.5%)	21 (9.6%)	34 (4.5%)
Fairly Ineffective	39 (10.5%)	57 (14.8%)	96 (12.8%)
Can't decide	159 (42.9%)	178 (46.4%)	337(44.9%)
Fairly effective	122 (32.9%)	87 (22.7)	209 (27.8%)
Very effective	38 (10.2%)	37 (9.6%)	75(10.0%)
Missing	0	4 (0.5%)	4 (0.5%)
Treatment Preference			
Acupuncture	196 (52.8%)	234 (60.9%)	430 (57.5%)
Counselling	94 (25.3%)	70 (18.2%)	164(21.9%)
Usual Care	3 (0.8%)	7 (1.8%)	10 (1.3%)
No preference	76 (20.5%)	68 (17.7%)	144 (19.3%)
Missing	2 (0.5%)	5 (1.3%)	7 (0.9%)

Do patients reporting pain at baseline have different outcomes in response to treatment?

Does baseline pain impact on depression at 3 months?

Using the average across all treatment groups, participants in the pain group showed less reduction in depression scores at three months compared to baseline (mean 16.70 to 12.06 at 3 months) than the no pain comparator group (baseline mean 14.06 to 9.10 at 3 months). A linear regression model found that the presence of moderate or extreme pain at baseline predicted a poorer outcome of depression treatment at 3 months (mean difference = -1.72, 95% CI: 9.13 to 10.43to $p<0.001$), whilst controlling for baseline depression scores. A series of regression models (supplement 1) identified three other significant predictors of poorer outcome of depression: poor EQ-5D mobility, loss of energy (BDI-II item 15) and being male. An ANCOVA

For peer review only

model controlling for these covariates and baseline PHQ-9 revealed that the effect of pain group remained significant, with patients with baseline pain having poorer depression outcomes (mean difference = -1.16, 95%CI: -2.2 to -0.12, p=0.028).

Following inspection of summary statistics, histograms and Q-Q plots, approximate normality was ascertained for all continuous variables. When entering all covariates considered into a correlation matrix, all correlations were below 0.5 (range 0.022 to 0.446). For analysis models that included multiple covariates, all tolerance values were greater than 0.1 (range 0.788 to 0.991) and VIF values were less than 10 (1.010 to 1.269), suggesting no evidence for collinearity between predictors.

Do depression scores change over time as a response to treatment for depression?

Figure 1 presents PHQ-9 depression scores by pain group and by trial arm at baseline and all follow up time points. Controlling for baseline depression and covariates, an ANCOVA model including a pain group by treatment interaction term(F(2,603)= 2.138, p<0.119) showed that in the pain group, participants showed a large reduction in depression with acupuncture at three months (mean reduction in PHQ-9 from baseline (6.0, 95%CI = 5.07 to 7.11), with smaller reductions associated with counselling (4.3, 95%CI = 3.3 to 5.4) and usual care (2.7, 95%CI = 1.50 to 4.06). In comparison, no notable differences were seen between treatment arms within the no pain comparator group. Figure 1 shows that after the initial reduction in depression from baseline to 3 months, depression scores tended to remain relatively stable during the 6 to 12 month follow up period in both the pain group and no pain comparator group.

In an ANCOVA model (controlling for baseline measures of depression, EQ-5D mobility, BDI-II loss of energy and sex) participants who received acupuncture reported significantly more depression free days between baseline to three months follow up than those receiving usual care alone (mean difference 15.2, 95%CI= 3.12 to 15.05). The differences between counselling and acupuncture, and between counselling and usual care were not significant. The no pain comparator group also showed a trend in favour of acupuncture. See Table2.

Table 2. Summary of depression free days at three months from baseline by pain group and trial arm.

	Depression free days		
	**No pain**	**Moderate –Extreme pain**	**Total**
**Treatment received**	Mean (Sd)	Mean (Sd)	Mean (Sd)
**Acupuncture**	37.3 (26.4)	31.3 (23.8)	31.7 (25.3)
**Counselling**	33.0 (22.2)	20.7 (21.9)	26.9 (22.9)
**Usual Care**	32.0 (23.5)	16.1(22.2)	22.6 (23.5)

Does bodily pain change over time as a response to treatment for depression?

Using the SF-36 bodily pain score at 3 months follow up as the endpoint and controlling for baseline SF-36 bodily pain and baseline PHQ-9 depression scores, results of ANCOVA show that the pain group continued to experience significantly worse pain after treatment for depression compared to the no pain comparator group (mean difference =14.57, 95% CI = 9.73 to 19.40). There was also a significant interaction between pain group and treatment arm (F(2,1) = 3.3, p=0.036 where pain group patients who received acupuncture for depression experienced a greater reduction in SF-36 bodily pain (represented by an increase in scores) between baseline to three months follow up (mean reduction = 11.2, 95% CI= 7.1 to 15.2), than those who received counselling (mean reduction = 7.6, 95% CI = 3.6 to 11.6) or usual care (mean reduction =7.2, 95% CI= 2.3 to 12.1). The reduction in pain at 3 months persisted through to the 12 month follow up point (Figure 2), however, the median of pain group after 12 months (median = 41, IQ1=31, IQ3 = 62) remained below the trial baseline median of 52 on the SF-36 bodily pain scale.

Adverse events

Treatment response may also be moderated by adverse events; 32% of participants in the pain group reported some form of adverse event between baseline and 12 months compared to 15% of the no pain comparator group, and were twice as likely to report an adverse event (OR = 2.05, 95%CI = 1.47 to 2.88).

DISCUSSION

Summary of results

For peer review only

Participants with moderate to severe pain at baseline had worse outcomes for depression than the no pain comparator group in all three treatment arms after controlling for baseline depression. The results of this sub-study confirm the main results of the ACUDep trial[15] by showing that at 3 months, both acupuncture and counselling interventions were effective for depression compared to usual care alone irrespective of the presence of comorbid pain. The results also extend the findings of the ACUDep trial[15] by showing that both acupuncture and counselling remain effective treatment options for patients with depression who also have comorbid pain. In addition, participants in the pain group had greater reductions in both depression symptoms with acupuncture from baseline to 3 months than those who received counselling or usual care. All treatment options were effective in reducing pain between baseline and 3 months follow up after controlling for baseline pain, however, acupuncture delivered a greater degree of pain relief than counselling or usual care in the short to medium term.

Strengths and Limitations

This study targets the under researched area of how pain may impact on the outcome of different treatments for depression. The research questions were clearly defined to establish the prevalence of pain in a depressed population, to describe the demographic profile of depressed people in pain compared to those who are depressed and pain free, to determine the impact of pain on the treatment outcomes, and in turn determine the effect of treatment for depression on the level of pain reported in the months subsequent to receiving treatment for depression. As a sub study of a larger pragmatic trial, the findings are important for three reasons; firstly the emphasis on external validity with findings that are generalizable to primary care patients with depression across the UK. Secondly, the method of randomisation to treatment allocation is a feature that was maintained through the sub-grouping into a ‘pain group’ and ‘no pain group’ to provide control for temporal effects and random factors that might influence the outcome of treatment. Finally, the standardised treatment protocols allowed for treatment to be tailored to individual patient’s needs regarding pain and depression whilst maintaining high standards of care, qualifications and practice experience. In terms of limitations, with regards to missing data; the patients who did not respond to follow up at three months were divided equally between the pain group and no pain group, and their baseline levels of depression were similar to those who did respond. This study was a sub study of a larger trial which was not powered to detect differences between the subgroups of moderate to extreme pain and no pain. The use of the BDI-II allowed for physical symptoms of depression to act as predictors to treatment outcome at three months follow up, however this outcome was not measured again at 3 months. Therefore, changes to factors such as sleeplessness, energy loss from within the BDI-II could not be assessed; however, these attributes will be explored qualitatively in a follow up paper. A further limitation is that the actual medication prescribed to participants and the frequency of use was not recorded at baseline or as follow up data, therefore the impact of the treatment on the types of anti-depressant and analgesic medication used or the frequency of use could not be established.

Relationship to literature

Incidence of comorbidity

In terms of comorbidity, the estimated prevalence of moderate to extreme pain within our study population of depressed patients is 51%, which is comparable with the 50-66% in previous literature. [1,2,6,24] This is a consequence of the ACUDep trial[15] targeting primary care patients with moderate to severe depression on the BDI-II. The doubling of adverse events reported by patients in moderate to extreme pain may be accounted for by attendance for hospital investigations and overnight admissions in relation to existing illnesses. However, patients with comorbid pain and depression tend to exhibit a cognitive bias specific to negative aspects of health[25] and are more likely to report less favourable outcomes of treatment.[26] Therefore, it is possible that treatment related minor adverse events are more likely to be reported by patients who suffer from chronic pain.

Predictors of depression

Evidence from the English longitudinal study of ageing[27] identified pain and mobility disability at baseline as predictors of comorbid pain and depression. In a large European study, a higher number of pain locations, pain of the joints and longer duration of pain (for 90 days or more), daily use of pain medication, and more severe pain at baseline were found to be associated with a significantly increased risk of still having a depressive or anxiety disorder after 2 years.[28]Together these factors are known to adversely affect the outcomes of treatment for depression.[7,8,29] Consistent with previous research, the majority of painful complaints within the study sample were of musculoskeletal origin and accompanied by poor mobility and a loss of energy. Pain due to osteoarthritis is known to determine subsequent depressed mood through its effect on fatigue and disability.[30]

Reduction in pain

The reductions in pain from baseline to 3 months are within the lower range of 5-30 points for a minimally clinically important difference[18,31], however, the majority of patients with pain remained below the trial baseline median of 52 on the SF-36 bodily pain scale at 3 months That patients reported reduced pain following acupuncture, is not surprising; 32% of patients had chronic muscular skeletal pain, for which there is a growing body of evidence supporting the efficacy of acupuncture.[11] With acupuncture being a holistic therapy, it is likely that acupuncturists incorporated treatment for pain alongside treatment for the symptoms of depression. Patients who received counselling also reported a more gradual reduction in pain over the 12 month follow up period. This finding is consistent with a Cochrane Review[31] which reports that psychological therapies, primarily CBT , can help people with people with chronic pain, reduce negative mood (depression and anxiety), disability, and to a lesser degree reduce pain over a six month period.

Implications for clinical practice and future research

Frequently patients with comorbid pain and depression will attribute their complaint to one or other of the conditions and seek help accordingly.[32] Patients presenting with pain or sleep disturbances are at risk of having a diagnosis of depression missed.[30] For treatment success, both pain and depression should be recognized and treated from the outset. The evidence emerging for the current study is that both acupuncture and counselling appear to have the potential to reduce symptoms associated with pain and depression concurrently, potentially affording patients' relief from symptoms of depression and reduced intensity of pain in both the short and longer term. This study provides a platform from which to develop a larger investigation specifically designed with sufficient power to determine the impact of acupuncture or counselling when compared to other treatments for depression in patients with comorbid pain and depression.

CONCLUSION

Patients who had moderate to extreme pain comorbid with depression at baseline recovered less well when compared to those who were pain free. Over three months, larger reductions in depression and pain scores were found in those who received acupuncture compared to those receiving counselling, and in turn these were greater than those receiving usual care. For those in pain at baseline, both acupuncture and counselling delivered a clinically meaningful reduction in bodily pain over 12 months.

For peer review only

Acknowledgements:
We would like to acknowledge the contribution of the patients, counsellors, acupuncturists and general medical practitioners; the ACUDep trial management team and members of MIND York.

Funding:
This article presents independent research funded by the National Institute for Health Research (NIHR) under its Programme Grants for Applied Research Programme (RP-PG-0707-10186). The views expressed are those of the author(s) and not necessarily those of the National Health Service, the NIHR or the Department of Health. The funders, and the University of York in its role as sponsor for the study, had no role in study design, data collection and analysis, decision to publish, or preparation of the manuscript.

Contributors:
Ann Hopton conducted the analysis, interpreted the data, drafted and revised the article.
Hugh MacPherson revised critically important intellectual content and gave final approval for publication.
Ada Keding provided substantial contribution to the conception and design and final approval for publication secondary analysis of the data and final approval for publication.
Stephen Morley provided substantial contribution to the conception and design, and final approval for publication.

Competing interests:
There are no competing interests.

Ethics approval:
Ethical approval was obtained within the ACUDEP trial (ISRCTN63787732) (REF) from the York NHS Research Ethics Committee (ref:09/H1311/75).

Data Sharing Statement: There are no additional unpublished data.

Figure legends:

Figure 1. PHQ-9 depression scores by pain/no pain groups and trial arm at baseline and follow up time points.

Figure 2. A summary of the SF-36 bodily pain scores by pain /no pain group and trial arm at baseline and follow up time points.

SUPPLEMENT 1.

A series of regression analyses to identify variables predicting poor outcome of treatment for depression at 3 months.

For peer review only

REFERENCES

1 Katona C, Peveler R, Dowrick C, *et al.* Pain symptoms in depression: definition and clinical significance. *Clinical Medicine* 2005;**5**:390–5. doi:10.7861/clinmedicine.5-4-390

2 Arnow BA. Comorbid Depression, Chronic Pain, and Disability in Primary Care. *Psychosomatic Medicine* 2006;**68**:262–8. doi:10.1097/01.psy.0000204851.15499.fc

3 Linton SJ, Nicholas MK, MacDonald S, *et al.* The role of depression and catastrophizing in musculoskeletal pain. *European Journal of Pain* 2011;**15**:416–22.

4 CG91: Depression in adults with a chronic physical health problem: Treatment and management.

5 Green A, Crawford A, Lewis G, *et al.* Are baseline physical symptoms characteristic of severe depression or a poor prognostic factor? Secondary analysis of the GenPod trial. *Journal of Psychosomatic Research* 2013;**74**:546. doi:10.1016/j.jpsychores.2013.03.038

6 Bair MJ, Robinson RL, Eckert GJ, *et al.* Impact of pain on depression treatment response in primary care. *PsychosomMed* 2004;**66(1)**:17–22.

7 Kroenke K, Shen J, Oxman TE, *et al.* Impact of pain on the outcomes of depression treatment: Results from the RESPECT trial. *Pain* 2008;**134**:209–15. doi:10.1016/j.pain.2007.09.021

8 DeVeaugh-Geiss AM, West SL, Miller WC, *et al.* The Adverse Effects of Comorbid Pain on Depression Outcomes in Primary Care Patients: Results from the Artist Trial. *Pain Medicine* 2010;**11**:732–41. doi:10.1111/j.1526-4637.2010.00830.x

9 Ennis E. Complementary and alternative medicines (CAMs) and adherence to mental health medications. *BMC Complementary and Alternative Medicine* 2014;**14**:93. doi:10.1186/1472-6882-14-93

10 Bergbom S, Boersma K, Overmeer T, *et al.* Relationship Among Pain Catastrophizing, Depressed Mood, and Outcomes Across Physical Therapy Treatments. *Physical Therapy* 2011;**91**:754–64. doi:10.2522/ptj.20100136

11 Vickers AJ. Acupuncture for Chronic Pain: Individual Patient Data Meta-analysis. *Archives of Internal Medicine* 2012;**172**:1444. doi:10.1001/archinternmed.2012.3654

12 Hopton AK, Curnoe S, Kanaan M, *et al.* Acupuncture in practice: mapping the providers, the patients and the settings in a national cross-sectional survey. *BMJ Open* 2012;**2**.http://bmjopen.bmj.com/content/2/1/e000456.abstract

13 Thomas KJ, Nicholl JP, Coleman P. Use and expenditure on complementary medicine in England: a population based survey. *Complement TherMed* 2001;**9**:2–11.

14 Mellor-Clark J, Simms-Ellis R, Burton M. National survey of counsellors working in primary care: evidence for growing professionalisation? *Occas Pap R Coll Gen Pract* 2001;**79**:vi–7.

15 MacPherson H, Richmond S, Bland M, *et al.* Acupuncture and Counselling for Depression in Primary Care: A Randomised Controlled Trial. *PLoS Medicine* 2013;**10**:e1001518. doi:10.1371/journal.pmed.1001518

16 MacPherson H, Richmond S, Bland MJ, *et al.* Acupuncture, Counseling, and Usual care for Depression (ACUDep): study protocol for a randomized controlled trial. *Trials* 2012;**13**:209.

For peer review only

17 Beck A, Ward C, Mendelson M, *et al.* An inventory for measuring depression. *ArchGenPsychiatry* 1961;**4**:561–71.

18 Garratt A, Ruta D, Abdalla M, *et al.* The SF36 health survey questionnaire: an outcome measure suitable for routine use within the NHS? *BMJ* 1993;**306**:1440–4.

19 MacPherson H, Elliot B, Hopton A, *et al.* Acupuncture for Depression: Patterns of Diagnosis and Treatment within a Randomised Controlled Trial. *Evidence-Based Complementary and Alternative Medicine* 2013;**2013**:1–9. doi:10.1155/2013/286048

20 Roth A, Hill A, Pilling S. The competences required to deliver effective Humanistic Psychological Therapies. *University College London, Centre for Outcomes Research and Effectiveness*;:2009.

21 Kroenke K, Spitzer RL, Williams JB. The PHQ-9: validity of a brief depression severity measure. *J Gen Intern Med* 2001;**16**:606–13.

22 Mischoulon D, Brill CD, Ameral VE, *et al.* A pilot study of acupuncture monotherapy in patients with major depressive disorder. *Journal of Affective Disorders* 2012;**141**:469–73. doi:10.1016/j.jad.2012.03.023

23 Vannoy SD, Arean P, Unutzer J. Advantages of using estimated depression-free days for evaluating treatment efficacy. *Psychiatr Serv* 2010;**61**:160–3.

24 Kroenke K, Wu J, Bair MJ, *et al.* Reciprocal Relationship Between Pain and Depression: A 12-Month Longitudinal Analysis in Primary Care. *The Journal of Pain* 2011;**12**:964–73. doi:10.1016/j.jpain.2011.03.003

25 Rusu AC, Pincus T, Morley S. Depressed pain patients differ from other depressed groups: Examination of cognitive content in a sentence completion task. *PAIN* 2012;**153**:1898–904. doi:10.1016/j.pain.2012.05.034

26 Ohayon MM, Schatzberg AF. Chronic pain and major depressive disorder in the general population. *Journal of Psychiatric Research* 2010;**44**:454–61. doi:10.1016/j.jpsychires.2009.10.013

27 Chou K-L. Combined effect of vision and hearing impairment on depression in older adults: Evidence from the English Longitudinal Study of Ageing. *Journal of Affective Disorders* 2008;**106**:191–6. doi:10.1016/j.jad.2007.05.028

28 Gerrits MMJG, Van Oppen P, Van Marwijk HWJ, *et al.* Pain and the onset of depressive and anxiety disorders. *PAIN®* 2014;**155**:53–9. doi:10.1016/j.pain.2013.09.005

29 Hawker GA, Gignac MAM, Badley E, *et al.* A longitudinal study to explain the pain-depression link in older adults with osteoarthritis. *Arthritis Care & Research* 2011;**63**:1382–90. doi:10.1002/acr.20298

30 White DK, Wilson JC, Keysor JJ. Measures of adult general functional status: SF-36 Physical Functioning Subscale (PF-10), Health Assessment Questionnaire (HAQ), Modified Health Assessment Questionnaire (MHAQ), Katz Index of Independence in Activities of Daily Living, Functional Independ. *Arthritis Care & Research* 2011;**63**:S297–S307. doi:10.1002/acr.20638

31 Eccleston C, Williams AC de C, Morley S. Psychological therapies for the management of chronic pain (excluding headache) in adults. In: The Cochrane Collaboration, Eccleston C, eds. *Cochrane Database of Systematic Reviews*. Chichester, UK: : John Wiley & Sons, Ltd 2009. http://doi.wiley.com/10.1002/14651858.CD007407.pub2 (accessed 5 Dec2013).

32 Robinson MJ, Edwards SE, Iyengar S, *et al.* Depression and pain. *Front Biosci (Landmark Ed)* 2009;**14**:5031–51.

For peer review only

ACUPUNCTURE, COUNSELLING OR USUAL CARE FOR DEPRESSION AND COMORBID PAIN: SECONDARY ANALYSIS OF A RANDOMISED CONTROLLED TRIAL

A. Hopton[1], H. MacPherson[1], A. Keding [1], S. Morley[2]

[1] Department of Health Sciences, University of York, UK

[2]Leeds Institute of Health Sciences - University of Leeds

Address for correspondence:

Ann Hopton
Research Fellow
Department of Health Sciences
University of York
Heslington
York YO10 5DD
United Kingdom
Tel: 01904 321384
E-mail: ann.hopton@york.ac.uk

Keywords: Depression, comorbid pain, acupuncture, counselling, usual care
Words 4,082.

For peer review only

ABSTRACT

Background
Depression with comorbid pain is associated with a poor response to various treatments. The objective in this secondary analysis is to determine whether patients reporting pain have different depression and pain outcomes over time in response to acupuncture, counselling or usual care.

Methods
Self-reported ratings of depression and pain from 755 patients in a pragmatic randomised controlled trial of acupuncture (302) or counselling (302) compared to usual care alone (151) are described and analysed using a series of regression models and ANCOVA. Patients reported outcomes of PHQ-9 for depression, SF36 bodily pain and EQ-5D, all at baseline, 3, 6, 9, and 12 months.

Results
At baseline, 755 patients reported EQ-5D pain categories; 384 (50.9%) reported moderate to extreme pain. Controlling for baseline depression, a linear regression model showed that the presence of pain at baseline was associated with poorer depression outcomes at 3 months mean difference = -1.16, (95%CI = 0.12 to 2.2) Participants with moderate to extreme pain at baseline did better at 3 months if they received acupuncture (mean reduction in PHQ-9 from baseline = 6.0, 95%CI = 5.0 to 7.1 and a mean reduction in SF-36 bodily pain = 11.2, (95% CI= 7.1 to 15.2) compared to improvements for those who received counselling (4.3, 95%CI = 3.3 to 5.4; 7.6, 95% CI = 3.6 to 11.6) or usual care (2.7, 95%CI = 1.50 to 4.0: 7.2, 95% CI= 2.3 to 12.1). In comparison, no notable differences were seen between treatment arms within the no pain comparator group.

Conclusion
Patients with depression and pain at baseline recovered less well from treatment over three months than those with depression and no pain. Reductions in both depression and pain were most marked in the acupuncture group, followed by the counselling group, and then the usual care group.

(299 words)

Strengths:

- This study targets the under researched area of how pain may impact on the outcome of different treatments for depression.
- The research questions were clearly defined to establish the prevalence of pain in a depressed population, to describe the demographic profile of depressed people in pain compared to those who are depressed and pain free, to determine the impact of pain on the treatment outcomes, and in turn determine the effect of treatment for depression on the level of pain reported in the months subsequent to receiving treatment for depression.
- As a sub study of a larger pragmatic trial, the findings are important for three reasons; firstly the emphasis on external validity with findings that are generalizable to primary care patients with depression across the UK. The method of randomisation to treatment allocation is a feature that was maintained through the sub-grouping into a 'pain group' and 'no pain group' to provide control for temporal effects and random factors that might influence the outcome of treatment.

Limitations:

- This study was a sub study of a larger trial which was not powered to detect differences between the subgroups of moderate to extreme pain and no pain.
- The use of the BDI-II outcome was not measured at 3 months.
- Changes to factors such as sleeplessness, energy loss from within the BDI-II could not be assessed at the primary outcome point.
- The name of the medication prescribed to participants and the frequency of use was not recorded at baseline or as follow up data, therefore the impact of the treatment on the types of anti-depressant and analgesic medication used or the frequency of use could not be established.

For peer review only

For peer review only - http://bmjopen.bmj.com/site/about/guidelines.xhtml

INTRODUCTION

Around 50%-66% of patients who are depressed also report pain.[1,2] Depression accentuates existing pain problems[3] adding to the emotional distress and poor sleep irrespective of whether the pain had a known cause[4]. Increased pain symptoms may also represent a greater severity of depression rather than a poor prognostic factor.[5] In terms of treatment, comorbid pain and depression are present in two thirds of depressed primary care patients who commence antidepressant therapy.[6] Pain is known to interfere with the routine activities in 42% of primary care patients prior to starting pharmacological treatment for depression[7] and has a strong negative impact on achieving remission.[8] Too frequently there is a focus on the treatment of either pain or depression. The use of complementary therapies alongside conventional treatments for depression is widespread; this uptake may be attributed to the perceptions of ineffectiveness to patients, the distress of continued symptoms and the unwanted side effects of medication [9].Treatment outcomes may be enhanced by taking an holistic approach, and delivering treatment options that address both pain and depression together.[10]

A meta-analysis of 18,000 participants data from 25 high quality trials has shown that acupuncture is an effective treatment option for several chronic pain conditions.[11] Acupuncture is also regularly provided by acupuncturists to treat depression[12] although not routinely available as a treatment option for depression within the UK's National Health Service.[13] Counselling for depression is widely available in primary care practices[14] however there is limited evidence for counselling compared to usual care as a treatment for patients with depression and a chronic physical health problem.[4,15] A recent randomised controlled trial of acupuncture or counselling provided to patients with depression found that both interventions significantly reduced depression at three months when compared to usual care.[15] Pain was a secondary outcome in this trial, but has not yet been reported, and it will be of interest to compare the treatment outcomes of patients who had comorbid pain and depression with those who had depression alone.

The primary aim of this study is to find out whether people with depression who are also in pain have better or worse depression outcomes than those without pain. In terms of objectives, we document the prevalence of pain in a depressed population, the demographic profile of patients in pain and the relationship between pain and depression. We then determine depression and pain outcomes following treatment with acupuncture or counselling when compared to usual care alone.

METHODS

Design

This research is a sub-study nested within a three-arm randomised controlled trial of acupuncture or counselling as adjuncts to usual care compared to usual care alone.[15] Full details of the ACUDep trial protocol are reported elsewhere.[15,16] Whilst maintaining the integrity of the 2:2:1 randomisation allocation of the ACUDep trial[16], for the purpose of this sub-study participants were divided into two groups according to their response to the EQ-5D pain statements at baseline: I have no pain or discomfort; I have moderate pain or discomfort; I have extreme pain or discomfort. People who reported either moderate or extreme pain were considered together as the "pain group", the remainder were assigned as a "no pain comparator group". A summary of the sub-group allocations and other demographic variables are presented in Table 1.

Participants

Recruited to the main ACUDep trial[15] were 755 patients aged 18 or over and diagnosed with depression or who had consulted for depression within the previous 30 months with a baseline score of 20 or above on the Beck Depression Inventory-II, which this scale classes as "moderate" to "severe" depression.[17] Patients were recruited from general practices in rural and urban areas of Yorkshire, County Durham and Northumberland. Bodily pain scores on the SF-36[18] and any chronic physical conditions or pre-existing illnesses were reported by patients at baseline. Patients' details were recorded by the York Trials Unit and randomised by computer-generated block randomisation, with block sizes of 5 and 10 by an investigator with no clinical attachment to the trial. An unequal allocation ratio of 2:2:1 to acupuncture, counselling and usual care provided groups of 302, 302 and 151 respectively.

Interventions

For peer review only

Within the main ACUDep trial[15] participants allocated to the acupuncture and counselling groups received the offer of up to 12 sessions on a weekly basis. The acupuncture intervention was performed according to a treatment protocol developed and agreed by acupuncture practitioners, which allowed for individualised treatment within a standardised theory-driven framework. The acupuncturists were members of the British Acupuncture Council with a minimum post qualification experience of three years, specific details of the intervention are published elsewhere[16] The treatment protocol for the counselling intervention was based on competences independently developed for Skills for Health within National Occupational Standards for Psychological Therapies.[20] The counsellors were members of the British Association for Counselling and Psychotherapy with a minimum of 400 hours post qualification experience and used a non-directive approach to help clients express feelings, clarify thoughts, and reframe difficulties. Usual care available to all participants throughout the trial included prescribed pharmaceutical and other interventions provided by NHS primary or secondary mental health services.

Outcome measures

The pain and depression outcome measures used for this sub-study were collected as part of the ACUDep trial[15]. These included the primary depression score as measured by the Patient Health Questionnaire (PHQ-9)[21], which scores each of the 9 DSM-IV criteria for depression as “0” (not at all) to “3” (nearly every day). The primary endpoint was at 3 months, with further follow up at 6, 9 and 12 months. The patients’ experience of the effect of the treatment received can also be expressed as depression free days[22,23] which is an approximate summary measure derived from the PHQ-9 cut off scores averaged over the period between measurements[15]. Patient reported pain measures were collected at baseline and at 3, 6, 9, and 12 months follow up using the SF-36 Bodily Pain sub-scale, which has a range 0-100, where a score of 100 indicated no pain, and the EQ-5D categories of no pain or moderate pain or severe pain. At baseline, patients also recorded their use of anti-depressants and analgesic medication, and their preferences and expectations of treatment.

Data analysis

Prevalence and demographic profile of patients with pain

Descriptive statistics for EQ-5D pain and SF-36 pain scores were calculated. Based on the EQ-5D response categories, patients were allocated to a ‘no pain’ and ‘moderate or extreme pain’ group, using the median SF-36 cut-off where EQ-5D data were missing. A descriptive analysis of the demographic profile of the pain group and no pain comparator group was conducted: The participants’ gender, average age of the sample population and pain groups population, their age on leaving education, the onset of their depression, the number of episodes they experienced, and the baseline scores of PHQ-9 and BDI-II baseline, SF-36 bodily pain score reported.

Pain and depression at baseline

ANOVA was used to compare the mean baseline PHQ-9 scores between the pain group and no pain comparator group. For the pain group alone, a Kendall’s Tau correlation was used to test the association between the baseline scores of the PHQ-9 and SF-36 bodily pain score. This test was considered the most appropriate given the possibility of tied scores between the PHQ-9 and SF-36 bodily pain.

Formatted: Highlight

Baseline pain and depression at three months

A series of regression models was applied to determine the influence of baseline pain in the presence of other demographic variables on the PHQ-9 depression outcome at 3 months, the primary endpoint of the trial. The first model predicted PHQ-9 at 3 months from the baseline EQ-5D Pain Group (‘pain’ or ‘no pain’), controlling for baseline PHQ-9 scores. Additional predictors of PHQ-9 depression at 3 months were identified by individual univariate analyses of demographic variables, the BDI-II depression items and the five EQ-5D items whilst controlling for PHQ-9 scores at baseline. For this scoping exercise, a less conservative level of significance was set at $p<0.1$ in order for potential covariates not to be missed and to maintain consistency with the methodology of the main ACUDep trial analysis. Any variables identified by univariate analysis were then included in a combined linear regression model, and any significant covariates were taken forward to the final model including pain grouping. Controlling for any remaining significant covariates of the final model ($p<0.05$) and baseline PHQ-9 depression scores, ANCOVA was used to test whether baseline pain affected treatment outcomes measured by the PHQ-9 score at three months.

Normality of continuous variables was assessed by inspection of histograms and Q-Q plots. All predictor variables included in the combined models were checked for collinearity in a correlation matrix and through assessment of tolerance and variance inflation factor (VIF) values.

Baseline pain and treatment outcomes (depression and pain)

An interaction term between treatment and pain group in the above ANCOVA model was used to establish whether patients in pain responded differently to the treatments with regards to their PHQ-9 depression at 3 months than patients reporting no pain. The analysis was repeated for the outcome of depression free days at three months, an approximate summary measure derived from PHQ-9 cut-off scores averaged over the period between measurements.[15,23] Descriptive analysis of the depression and pain scores was conducted over the 12 month follow up period.

Adverse events

A comparison of the adverse events reported between baseline and 12 month follow up was conducted using proportions and odds ratio.

Missing data

Formatted: Highlight

Results of the main ACUDep analysis revealed an average response rate of 81% for the PHQ-9 at 3 months follow-up, and the primary analysis employed multiple imputation by chained regression using selected demographics and baseline pain and depression outcomes to impute PHQ-9 scores. For the present secondary analysis, imputation was not considered. However, for the initial allocation of patients into 'pain' and 'no pain' sub-groups, the SF-36 bodily pain scale was used where the baseline EQ-5D pain item was not available in order to utilise all available outcome data. Outcomes were assumed to be missing at random, and the analysis of patients in their randomly allocated treatment groups (intention to treat) aimed to control for any random factors that might have influenced the outcome of treatment.

RESULTS

An exploration of pain and depression at baseline

What is the prevalence of pain?

Patients reported pain or discomfort on the EQ-5D questionnaire at baseline; 371 (49.1%) reported no pain or discomfort, 298 (39.5%) reported moderate pain or discomfort and 86 (11.4%) reported extreme pain or discomfort. The distribution of SF-36 bodily pain scores was negatively skewed; 140 (18.5%) patients reported a score of 100, indicating no pain at all, and the sample returned a median pain score of 52 (Interquartile 1= 25, Interquartile 3 = 84).

Allocation to "pain" and "no-pain" sub-groups

For the purpose of this study, those reporting moderate and extreme pain or discomfort on the EQ-5D questionnaire were merged together to form a single 'pain group' (n=384, 51%) with the remainder forming the 'no pain' comparator group (n=371, 49%). Patients who omitted to answer the EQ-5D pain question at baseline (n=7; 1%) were assigned to a group according to their SF-36 bodily pain score; 3 scored above the 3rd interquartile range of the SF-36 bodily pain scale and were assigned to the no pain group, the remaining four patients at baseline scored below the median and were assigned to the pain group. Within the defined pain and no-pain subgroups, trial arm allocations retained the 2:2:1 ratio of the original ACUDep[15] randomisation process.

What is the demographic profile of patients in pain and not in pain?

A summary of the demographic profiles, presented in Table 1, show that the pain and the no-pain groups were comparable for most baseline variables. Notable exceptions were: The pain group members tended to be older than the no pain comparator group (mean of 47 years, vs. 40 years) a difference which remained notable after temporarily removing ten pain group members with outlying ages between of 76-93 years. In terms of health and employment, 56% of the pain group (vs. 9% of the no-pain group) reported a painful health condition or illness that predated the onset of depression, for which 64% (vs. 31%) used analgesic medication regularly, 32% (vs. 9%) were unable to work or retired.

What is the relationship between pain and depression at baseline?

The baseline PHQ-9 depression scores indicate that the pain group reported higher levels of depression at baseline (mean PHQ-9=17.0, SD 5.2) than the no pain comparator group (mean PHQ-9=14.9, SD 5.2). Results of ANOVA confirmed the difference to be highly significant (mean difference 2.02, 95%CI =1.28 to 2.76). For the pain group alone the correlation between the PHQ-9 scores and SF-36 bodily pain scores was weak, but highly significant (Kendall's Tau -0.172, p<0.001).

For peer review only

Table 1. Demographics and variables of interest at baseline			
Characteristic	No Pain N= 371 (49%)	Moderate-extreme pain N= 384 (51%)	Total N=755 (100%)
Age			
Mean (SD)	39.9 (11.58)	46.83 (14.11)	43.5 (13.37)
Median (min, max)	39 (18,75)	46 (18,93)	43 (18,93)
IQR (25%, 75%)	31,48	38,56	33,53
Missing	-	-	-
Sex			
Male	88 (23.7%)	113(29.4%)	201 (26.6%)
Female	283 (76.3%)	271(70.6%)	554(73.4%)
Missing	-	-	-
Age left education			
Mean (SD)	18.3 (3.90)	17.53 (4.61)	18.0 (4.37)
Median (min, max)	17.0 (13, 52)	16 (14, 54)	16 (13-54)
IQR (25%, 75%)	16, 21	16,18	16,19
Missing	-	-	-
Employment			
Full-time education	13 (3.5%)	10 (2.6%)	23 (3.1%)
Working full-time	167 (45%)	114 (29.7%)	281 (37%)
Working part-time	83 (22.4%)	61 (15.9%)	144 (19.5%)
Unable to work	19 (5.1%)	76 (19.8%)	95 (12.9%)
Looking after home	41 (11.1%)	42 (10.9%)	83 (11%)
Retired	14 (3.8%)	51 (13.3%)	65(8.8%)
Other	26 (7%)	22 (5.7%)	48(6.5%)
Missing	8 (2.1 %)	8 (2.1%)	16(2.1%)
Painful health or medical condition			
Current painful health or medical condition	41(12.7%)	299 (77.9%)	346 (45.8%)
Onset before depression	32(8.6%)	216 (56.2%)	248 (32.8%)
Missing	2(0.5%)	4(1%)	6 (0.8%)
Type of health problem			
Musculoskeletal	21(6%)	221(58%)	242(32%)
Other	27(7%)	81 (21%)	108(14%)
No health problem	321(87%)	81 (21%	402 (53%)
SF-36 Bodily pain			
Mean (SD)	76.97(22.53)	39.03(22.5)	57.6 (28.44)
Median (min, max)	77.5 (12, 100)	41 (0, 100)	52 (0, 100)
IQR (25%, 75%)	62, 100	22, 51	32, 84
Missing	3 (0.39%)	1 (1.3%)	4 (0.5%)
Depression			
In last 2 weeks	264 (71.2%)	310 (80.7%)	574 (76.1%)
Missing	2 (0.5%)	10 (2.6%)	12 (1.6%)
Not first major episode	240(64.7%)	273 (71.1%)	513 (67.9%)
Missing	108 (29.1%)	82 (21.3%)	190 (25.2%)
4+ previous episodes	162 (43.7%)	227 (59.1%)	389 (51.5%)
Missing	133 (35.8%)	113 (29.4%)	246 (32.6%)
Age at first major depressive episode			
Mean (SD)	24.89 (11.56)	25.47 (12.9)	25.2 (12-28)
Median (min, max)	21 (6, 72)	23 (0-79)	22(0-79)
IQR (25%, 75%)	16, 32	16, 31	16,31
Missing	7 (0.9%)	9(1.2%)	16 (2.1%)
Medication			
Depression medication in last 3 months	250 (67.3%)	269 (70%)	519 (68.7%)
Missing	-	-	-
Analgesic medication in last 3 months	114 (30.7%)	245 (63.8%)	359 (47.5%)
Missing	4 (1%)	2 (0.52 %)	6 (0.8%)
EQ-5D Pain			
No pain	371 (49.1%)	0	371 (49.1%)
Moderate pain or discomfort	0	298 (77.6%)	298 (39.5%)
Extreme pain or discomfort	0	86 (22.4%)	86 (11.4%)
Missing	3	4	7 (0.9%)

Table 1. Demographics and variables of interest at baseline			
Characteristic	No Pain N= 371 (49%)	Moderate-extreme pain N= 384 (51%)	Total N=755 (100%)
EQ-5D Anxiety/Depression			
Not anxious/depressed	13 (3.5%)	8 (2.1%)	21 (2.8%)
Moderately anxious/depressed	282 (76%)	272 (70.8%)	554 (73.4%)
Extremely anxious/depressed	74 (19%)	104 (27%)	178 (23.6%)
Missing	2 (0.5%)	0	2 (0.3%)
EQ5D Mobility			
No problems walking about	361 (31%)	234 (31%)	595 (79%)
Some problems walking about	9(1.2%)	148(19.7%)	157(20.8%
Confined to bed	0(0%)	1(0.1%)	1(0.1%)
Missing	-	-	-
EQ5D Self-Care			
No problems with self-care	358 (47.5%)	314 (41.7%)	672 (89.2%)
Some problems with self-care	11(1.5%)	68 (9%)	79 (10.5%)
Unable to wash or dress	1(0.1%)	1 (0.1%)	2 (0.3%)
Missing	1(0.1%)	1(0.1%)	2(0.3%)
EQ5D Usual activities			
No problems performing usual activities	251(33.2%)	111(14.7%)	362(48.1%)
Some problems performing usual activities	115(15.3%)	247(32.8%)	115(48.1%)
Unable to perform usual activities	5 (0.7%)	24 (3.2%)	29(3.9%)
Missing	2(0.3%)	0(0%)	2(0.3%)
PHQ-9			
Mean (SD)	14.97 (5.15)	17.0 (5.23)	16.0 (5.29)
Median (min, max)	15 (3, 27)	17 (3, 27)	16 (3-27)
IQR (25%, 75%)	11, 19	13, 21	12, 20
Missing	-	1 (0.2%)	1 (0.1%)
BDI-II			
Mean (SD)	31.32 (8.29)	33.6 (8.9)	32.5 (8.72)
BDI-II Group			
Moderate (20-28)	155 (41.8%)	129 (33.6%)	284 (37.6%)
Severe (29-63)	216 (52.8%)	255 (66.4%)	471 (62.4%)
Trial arm allocation			
Acupuncture	156 (20.7%)	146 (19.3%)	302(40%
Counselling	151 (20%)	151 (20%)	302(40%)
Usual Care	64 (8.5%)	87 (11.5%)	151(20%)
Missing	-	-	-
Expectation of treatment allocated			
Very Ineffective	13 (3.5%)	21 (9.6%)	34 (4.5%)
Fairly Ineffective	39 (10.5%)	57 (14.8%)	96 (12.8%)
Can't decide	159 (42.9%)	178 (46.4%)	337(44.9%)
Fairly effective	122 (32.9%)	87 (22.7)	209 (27.8%)
Very effective	38 (10.2%)	37 (9.6%)	75(10.0%)
Missing	0	4 (0.5%)	4 (0.5%)
Treatment Preference			
Acupuncture	196 (52.8%)	234 (60.9%)	430 (57.5%)
Counselling	94 (25.3%)	70 (18.2%)	164(21.9%)
Usual Care	3 (0.8%)	7 (1.8%)	10 (1.3%)
No preference	76 (20.5%)	68 (17.7%)	144 (19.3%)
Missing	2 (0.5%)	5 (1.3%)	7 (0.9%)

Do patients reporting pain at baseline have different outcomes in response to treatment?

Formatted: Highlight

Does baseline pain impact on depression at 3 months?

Using the average across all treatment groups, participants in the pain group showed less reduction in depression scores at three months compared to baseline (mean 16.70 to 12.06 at 3 months) than the no pain comparator group (baseline mean 14.06 to 9.10 at 3 months). A linear regression model found that the presence of moderate or extreme pain at baseline predicted a poorer outcome of depression treatment at 3 months (mean difference = -1.72, 95% CI: 9.13 to 10.43to $p<0.001$), whilst controlling for baseline depression scores. A series of regression models (supplement 1) identified three other significant predictors of poorer outcome of depression: poor EQ-5D mobility, loss of energy (BDI-II item 15) and being male. An ANCOVA

For peer review only

model controlling for these covariates and baseline PHQ-9 revealed that the effect of pain group remained significant, with patients with baseline pain having poorer depression outcomes (mean difference = -1.16, 95%CI: -2.2 to -0.12, p=0.028).
Following inspection of summary statistics, histograms and Q-Q plots, approximate normality was ascertained for all continuous variables. When entering all covariates considered into a correlation matrix, all correlations were below 0.5 (range 0.022 to 0.446). For analysis models that included multiple covariates, all tolerance values were greater than 0.1 (range 0.788 to 0.991) and VIF values were less than 10 (1.010 to 1.269), suggesting no evidence for collinearity between predictors.

Do depression scores change over time as a response to treatment for depression?
Figure 1 presents PHQ-9 depression scores by pain group and by trial arm at baseline and all follow up time points. Controlling for baseline depression and covariates, an ANCOVA model including a pain group by treatment interaction term(F(2,603)= 2.138, p<0.119) showed that in the pain group, participants showed a large reduction in depression with acupuncture at three months (mean reduction in PHQ-9 from baseline (6.0, 95%CI = 5.07 to 7.11), with smaller reductions associated with counselling (4.3, 95%CI = 3.3 to 5.4) and usual care (2.7, 95%CI = 1.50 to 4.06). In comparison, no notable differences were seen between treatment arms within the no pain comparator group. Figure 1 shows that after the initial reduction in depression from baseline to 3 months, depression scores tended to remain relatively stable during the 6 to 12 month follow up period in both the pain group and no pain comparator group.

Formatted: Highlight

In an ANCOVA model (controlling for baseline measures of depression, EQ-5D mobility, BDI-II loss of energy and sex) participants who received acupuncture reported significantly more depression free days between baseline to three months follow up than those receiving usual care alone (mean difference 15.2, 95%CI= 3.12 to 15.05). The differences between counselling and acupuncture, and between counselling and usual care were not significant. The no pain comparator group also showed a trend in favour of acupuncture. See Table2.

Table 2. Summary of depression free days at three months from baseline by pain group and trial arm.

	Depression free days		
	**No pain**	**Moderate –Extreme pain**	**Total**
**Treatment received**	Mean (Sd)	Mean (Sd)	Mean (Sd)
**Acupuncture**	37.3 (26.4)	31.3 (23.8)	31.7 (25.3)
**Counselling**	33.0 (22.2)	20.7 (21.9)	26.9 (22.9)
**Usual Care**	32.0 (23.5)	16.1(22.2)	22.6 (23.5)

Does bodily pain change over time as a response to treatment for depression?
Using the SF-36 bodily pain score at 3 months follow up as the endpoint and controlling for baseline SF-36 bodily pain and baseline PHQ-9 depression scores, results of ANCOVA show that the pain group continued to experience significantly worse pain after treatment for depression compared to the no pain comparator group (mean difference =14.57, 95% CI = 9.73 to 19.40). There was also a significant interaction between pain group and treatment arm (F(2,1) = 3.3, p=0.036 where pain group patients who received acupuncture for depression experienced a greater reduction in SF-36 bodily pain (represented by an increase in scores) between baseline to three months follow up (mean reduction = 11.2, 95% CI= 7.1 to 15.2), than those who received counselling (mean reduction = 7.6, 95% CI = 3.6 to 11.6) or usual care (mean reduction =7.2, 95% CI= 2.3 to 12.1). The reduction in pain at 3 months persisted through to the 12 month follow up point (Figure 2), however, the median of pain group after 12 months (median = 41, IQ1=31, IQ3 = 62) remained below the trial baseline median of 52 on the SF-36 bodily pain scale.

Adverse events
Treatment response may also be moderated by adverse events; 32% of participants in the pain group reported some form of adverse event between baseline and 12 months compared to 15% of the no pain comparator group, and were twice as likely to report an adverse event (OR = 2.05, 95%CI = 1.47 to 2.88).

DISCUSSION

Summary of results

For peer review only

Participants with moderate to severe pain at baseline had worse outcomes for depression than the no pain comparator group in all three treatment arms after controlling for baseline depression. The results of this sub-study confirm the main results of the ACUDep trial[15] by showing that at 3 months, both acupuncture and counselling interventions were effective for depression compared to usual care alone irrespective of the presence of comorbid pain. The results also extend the findings of the ACUDep trial[15] by showing that both acupuncture and counselling remain effective treatment options for patients with depression who also have comorbid pain. In addition, participants in the pain group had greater reductions in both depression symptoms with acupuncture from baseline to 3 months than those who received counselling or usual care. All treatment options were effective in reducing pain between baseline and 3 months follow up after controlling for baseline pain, however, acupuncture delivered a greater degree of pain relief than counselling or usual care in the short to medium term.

Strengths and Limitations

This study targets the under researched area of how pain may impact on the outcome of different treatments for depression. The research questions were clearly defined to establish the prevalence of pain in a depressed population, to describe the demographic profile of depressed people in pain compared to those who are depressed and pain free, to determine the impact of pain on the treatment outcomes, and in turn determine the effect of treatment for depression on the level of pain reported in the months subsequent to receiving treatment for depression. As a sub study of a larger pragmatic trial, the findings are important for three reasons; firstly the emphasis on external validity with findings that are generalizable to primary care patients with depression across the UK. Secondly, the method of randomisation to treatment allocation is a feature that was maintained through the sub-grouping into a 'pain group' and 'no pain group' to provide control for temporal effects and random factors that might influence the outcome of treatment. Finally, the standardised treatment protocols allowed for treatment to be tailored to individual patient's needs regarding pain and depression whilst maintaining high standards of care, qualifications and practice experience. In terms of limitations, with regards to missing data; the patients who did not respond to follow up at three months were divided equally between the pain group and no pain group, and their baseline levels of depression were similar to those who did respond. This study was a sub study of a larger trial which was not powered to detect differences between the subgroups of moderate to extreme pain and no pain. The use of the BDI-II allowed for physical symptoms of depression to act as predictors to treatment outcome at three months follow up, however this outcome was not measured again at 3 months. Therefore, changes to factors such as sleeplessness, energy loss from within the BDI-II could not be assessed; however, these attributes will be explored qualitatively in a follow up paper. A further limitation is that the actual medication prescribed to participants and the frequency of use was not recorded at baseline or as follow up data, therefore the impact of the treatment on the types of anti-depressant and analgesic medication used or the frequency of use could not be established.

Formatted: Highlight

Relationship to literature

Incidence of comorbidity

In terms of comorbidity, the estimated prevalence of moderate to extreme pain within our study population of depressed patients is 51%, which is comparable with the 50-66% in previous literature. [1,2,6,24] This is a consequence of the ACUDep trial[15] targeting primary care patients with moderate to severe depression on the BDI-II. The doubling of adverse events reported by patients in moderate to extreme pain may be accounted for by attendance for hospital investigations and overnight admissions in relation to existing illnesses. However, patients with comorbid pain and depression tend to exhibit a cognitive bias specific to negative aspects of health[25] and are more likely to report less favourable outcomes of treatment.[26] Therefore, it is possible that treatment related minor adverse events are more likely to be reported by patients who suffer from chronic pain.

Predictors of depression

Evidence from the English longitudinal study of ageing[27] identified pain and mobility disability at baseline as predictors of comorbid pain and depression. In a large European study, a higher number of pain locations, pain of the joints and longer duration of pain (for 90 days or more), daily use of pain medication, and more severe pain at baseline were found to be associated with a significantly increased risk of still having a depressive or anxiety disorder after 2 years.[29]Together these factors are known to adversely affect the outcomes of treatment for depression.[7,8,29] Consistent with previous research, the majority of painful complaints within the study sample were of musculoskeletal origin and accompanied by poor mobility and a loss of energy. Pain due to osteoarthritis is known to determine subsequent depressed mood through its effect on fatigue and disability.[30]

Formatted: Highlight

For peer review only

Reduction in pain

The reductions in pain from baseline to 3 months are within the lower range of 5-30 points for a minimally clinically important difference[18,31], however, the majority of patients with pain remained below the trial baseline median of 52 on the SF-36 bodily pain scale at 3 months That patients reported reduced pain following acupuncture, is not surprising; 32% of patients had chronic muscular skeletal pain, for which there is a growing body of evidence supporting the efficacy of acupuncture.[11] With acupuncture being a holistic therapy, it is likely that acupuncturists incorporated treatment for pain alongside treatment for the symptoms of depression. Patients who received counselling also reported a more gradual reduction in pain over the 12 month follow up period. This finding is consistent with a Cochrane Review[31] which reports that psychological therapies, primarily CBT , can help people with people with chronic pain, reduce negative mood (depression and anxiety), disability, and to a lesser degree reduce pain over a six month period.

Implications for clinical practice and future research

Frequently patients with comorbid pain and depression will attribute their complaint to one or other of the conditions and seek help accordingly.[32] Patients presenting with pain or sleep disturbances are at risk of having a diagnosis of depression missed.[30] For treatment success, both pain and depression should be recognized and treated from the outset. The evidence emerging for the current study is that both acupuncture and counselling appear to have the potential to reduce symptoms associated with pain and depression concurrently, potentially affording patients' relief from symptoms of depression and reduced intensity of pain in both the short and longer term. This study provides a platform from which to develop a larger investigation specifically designed with sufficient power to determine the impact of acupuncture or counselling when compared to other treatments for depression in patients with comorbid pain and depression.

CONCLUSION

Patients who had moderate to extreme pain comorbid with depression at baseline recovered less well when compared to those who were pain free. Over three months, larger reductions in depression and pain scores were found in those who received acupuncture compared to those receiving counselling, and in turn these were greater than those receiving usual care. For those in pain at baseline, both acupuncture and counselling delivered a clinically meaningful reduction in bodily pain over 12 months.

Figure legends:

Figure 1. PHQ-9 depression scores by pain/no pain groups and trial arm at baseline and follow up time points.

Figure 2. A summary of the SF-36 bodily pain scores by pain /no pain group and trial arm at baseline and follow up time points.

Acknowledgements:

We would like to acknowledge the contribution of the patients, counsellors, acupuncturists and general medical practitioners; the ACUDep trial management team and members of MIND York.

Funding:

This article presents independent research funded by the National Institute for Health Research (NIHR) under its Programme Grants for Applied Research Programme (RP-PG-0707-10186). The views expressed are those of the author(s) and not necessarily those of the National Health Service, the NIHR or the Department of Health. The funders, and the University of York in its role as sponsor for the study, had no role in study design, data collection and analysis, decision to publish, or preparation of the manuscript.

Competing interests:

For peer review only

There are no competing interests.

Ethics approval:
Ethical approval was obtained within the ACUDEP trial (ISRCTN63787732) (REF) from the York NHS Research Ethics Committee (ref:09/H1311/75).

Contributors:
Ann Hopton conducted the analysis, interpreted the data, drafted and revised the article.
Hugh MacPherson revised critically important intellectual content and gave final approval for publication.
Ada Keding provided substantial contribution to the conception and design and final approval for publication secondary analysis of the data and final approval for publication.
Stephen Morley provided substantial contribution to the conception and design, and final approval for publication.

REFERENCES

1 Katona C, Peveler R, Dowrick C, *et al.* Pain symptoms in depression: definition and clinical significance. *Clinical Medicine* 2005;**5**:390–5. doi:10.7861/clinmedicine.5-4-390

2 Arnow BA. Comorbid Depression, Chronic Pain, and Disability in Primary Care. *Psychosomatic Medicine* 2006;**68**:262–8. doi:10.1097/01.psy.0000204851.15499.fc

3 Linton SJ, Nicholas MK, MacDonald S, *et al.* The role of depression and catastrophizing in musculoskeletal pain. *European Journal of Pain* 2011;**15**:416–22.

4 CG91: Depression in adults with a chronic physical health problem: Treatment and management.

5 Green A, Crawford A, Lewis G, *et al.* Are baseline physical symptoms characteristic of severe depression or a poor prognostic factor? Secondary analysis of the GenPod trial. *Journal of Psychosomatic Research* 2013;**74**:546. doi:10.1016/j.jpsychores.2013.03.038

6 Bair MJ, Robinson RL, Eckert GJ, *et al.* Impact of pain on depression treatment response in primary care. *PsychosomMed* 2004;**66(1)**:17–22.

7 Kroenke K, Shen J, Oxman TE, *et al.* Impact of pain on the outcomes of depression treatment: Results from the RESPECT trial. *Pain* 2008;**134**:209–15. doi:10.1016/j.pain.2007.09.021

8 DeVeaugh-Geiss AM, West SL, Miller WC, *et al.* The Adverse Effects of Comorbid Pain on Depression Outcomes in Primary Care Patients: Results from the Artist Trial. *Pain Medicine* 2010;**11**:732–41. doi:10.1111/j.1526-4637.2010.00830.x

9 Ennis E. Complementary and alternative medicines (CAMs) and adherence to mental health medications. *BMC Complementary and Alternative Medicine* 2014;**14**:93. doi:10.1186/1472-6882-14-93

10 Bergbom S, Boersma K, Overmeer T, *et al.* Relationship Among Pain Catastrophizing, Depressed Mood, and Outcomes Across Physical Therapy Treatments. *Physical Therapy* 2011;**91**:754–64. doi:10.2522/ptj.20100136

11 Vickers AJ. Acupuncture for Chronic Pain: Individual Patient Data Meta-analysis. *Archives of Internal Medicine* 2012;**172**:1444. doi:10.1001/archinternmed.2012.3654

12 Hopton AK, Curnoe S, Kanaan M, *et al.* Acupuncture in practice: mapping the providers, the patients and the settings in a national cross-sectional survey. *BMJ Open* 2012;**2**.http://bmjopen.bmj.com/content/2/1/e000456.abstract

13 Thomas KJ, Nicholl JP, Coleman P. Use and expenditure on complementary medicine in England: a population based survey. *Complement TherMed* 2001;**9**:2–11.

For peer review only

14 Mellor-Clark J, Simms-Ellis R, Burton M. National survey of counsellors working in primary care: evidence for growing professionalisation? *Occas Pap R Coll Gen Pract* 2001;**79**:vi–7.

15 MacPherson H, Richmond S, Bland M, *et al.* Acupuncture and Counselling for Depression in Primary Care: A Randomised Controlled Trial. *PLoS Medicine* 2013;**10**:e1001518. doi:10.1371/journal.pmed.1001518

16 MacPherson H, Richmond S, Bland MJ, *et al.* Acupuncture, Counseling, and Usual care for Depression (ACUDep): study protocol for a randomized controlled trial. *Trials* 2012;**13**:209.

17 Beck A, Ward C, Mendelson M, *et al.* An inventory for measuring depression. *ArchGenPsychiatry* 1961;**4**:561–71.

18 Garratt A, Ruta D, Abdalla M, *et al.* The SF36 health survey questionnaire: an outcome measure suitable for routine use within the NHS? *BMJ* 1993;**306**:1440–4.

19 MacPherson H, Elliot B, Hopton A, *et al.* Acupuncture for Depression: Patterns of Diagnosis and Treatment within a Randomised Controlled Trial. *Evidence-Based Complementary and Alternative Medicine* 2013;**2013**:1–9. doi:10.1155/2013/286048

20 Roth A, Hill A, Pilling S. The competences required to deliver effective Humanistic Psychological Therapies. *University College London, Centre for Outcomes Research and Effectiveness*;:2009.

21 Kroenke K, Spitzer RL, Williams JB. The PHQ-9: validity of a brief depression severity measure. *J Gen Intern Med* 2001;**16**:606–13.

22 Mischoulon D, Brill CD, Ameral VE, *et al.* A pilot study of acupuncture monotherapy in patients with major depressive disorder. *Journal of Affective Disorders* 2012;**141**:469–73. doi:10.1016/j.jad.2012.03.023

23 Vannoy SD, Arean P, Unutzer J. Advantages of using estimated depression-free days for evaluating treatment efficacy. *Psychiatr Serv* 2010;**61**:160–3.

24 Kroenke K, Wu J, Bair MJ, *et al.* Reciprocal Relationship Between Pain and Depression: A 12-Month Longitudinal Analysis in Primary Care. *The Journal of Pain* 2011;**12**:964–73. doi:10.1016/j.jpain.2011.03.003

25 Rusu AC, Pincus T, Morley S. Depressed pain patients differ from other depressed groups: Examination of cognitive content in a sentence completion task. *PAIN* 2012;**153**:1898–904. doi:10.1016/j.pain.2012.05.034

26 Ohayon MM, Schatzberg AF. Chronic pain and major depressive disorder in the general population. *Journal of Psychiatric Research* 2010;**44**:454–61. doi:10.1016/j.jpsychires.2009.10.013

27 Chou K-L. Combined effect of vision and hearing impairment on depression in older adults: Evidence from the English Longitudinal Study of Ageing. *Journal of Affective Disorders* 2008;**106**:191–6. doi:10.1016/j.jad.2007.05.028

28 Gerrits MMJG, Van Oppen P, Van Marwijk HWJ, *et al.* Pain and the onset of depressive and anxiety disorders. *PAIN®* 2014;**155**:53–9. doi:10.1016/j.pain.2013.09.005

29 Hawker GA, Gignac MAM, Badley E, *et al.* A longitudinal study to explain the pain-depression link in older adults with osteoarthritis. *Arthritis Care & Research* 2011;**63**:1382–90. doi:10.1002/acr.20298

30 White DK, Wilson JC, Keysor JJ. Measures of adult general functional status: SF-36 Physical Functioning Subscale (PF-10), Health Assessment Questionnaire (HAQ), Modified Health Assessment Questionnaire (MHAQ), Katz Index of Independence in Activities of Daily Living, Functional Independ. *Arthritis Care & Research* 2011;**63**:S297–S307. doi:10.1002/acr.20638

For peer review only

31 Eccleston C, Williams AC de C, Morley S. Psychological therapies for the management of chronic pain (excluding headache) in adults. In: The Cochrane Collaboration, Eccleston C, eds. *Cochrane Database of Systematic Reviews*. Chichester, UK: : John Wiley & Sons, Ltd 2009. http://doi.wiley.com/10.1002/14651858.CD007407.pub2 (accessed 5 Dec2013).

32 Robinson MJ, Edwards SE, Iyengar S, *et al.* Depression and pain. *Front Biosci (Landmark Ed)* 2009;**14**:5031–51.

For peer review only

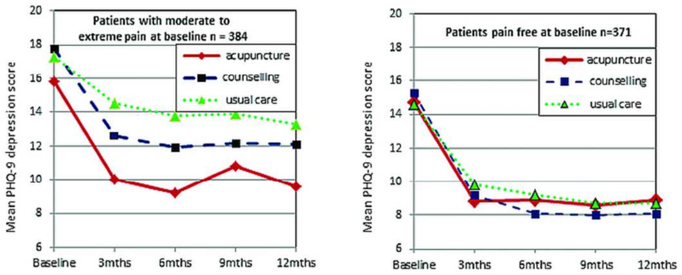

Figure 1. PHQ-9 depression scores by pain/no pain groups and trial arm at baseline and follow up time points.
90x67mm (300 x 300 DPI)

For peer review only

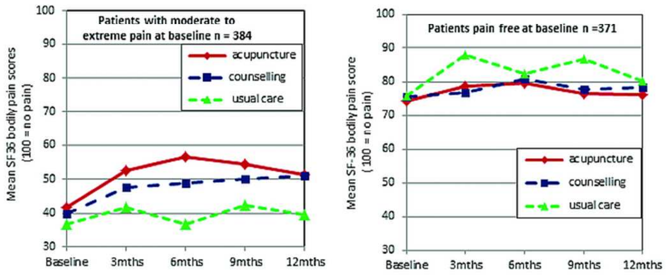

Figure 2. A summary of the SF-36 bodily pain scores by pain /no pain group and trial arm at baseline and follow up time points.
90x67mm (300 x 300 DPI)

For peer review only

SUPPLEMENT 1.
A series of regression analyses to identify variables predicting poor outcome of treatment for depression at 3 months.

Table 3. Univariate analyses of potential predictors of PHQ-9 depression at 3 months, controlling for baseline PHQ-9 (NB *denotes p<0.10)

Demographic Items	Beta	Std Error	95% Confidence interval	Sig p
Age	-0.003	0.018	-.036 to 0.33	0.853
Sex*	-1.076	0.522	-2.10 to 0.05	0.040
Age left education	-0.027	0.055	-0.13 to 0.82	0.633
Employment*	-0.377	0.113	0.15 to 0.59	0.001
Painful health or medical condition*	1.597	0.462	0.68 to 2.5	0.001
N Episodes of depression	0.056	0.139	-0.21 to 0.32	0.685
Age at onset of depression	-0.008	0.019	0.04 to 0.28	0.653
Medication- Analgesics*	-0.005	0.008	-0.10 to 0.01	0.115
Medication - Antidepressants	0.800	0.501	-0.18 to 1.78	0.111
Expectations of allocated treatment	0.701	0.273	-0.16 to 1.23	0.11
Preference for treatment	0.005	0.003	-0.11 to 0.11	0.710
**BDI-II Items**				
1. Sadness	0.223	0.389	-0.54 to 0.98	0.566
2.Pessimism	0.164	0.308	-0.44 to 0.76	0.594
3.Past Failure	0.618	0.316	-0.002 to 1.23	0.510
4.Loss of Pleasure	0.164	0.366	-0.55 to 0.88	0.654
5.Guilty feelings	0.262	0.278	-0.28 to 0.80	0.346
6.Punishment feelings	0.310	0.206	-0.94 to 0.71	0.133
7.Self-Dislike	0.415	0.281	-0.13 to 0.96	0.141
8.Self Criticalness	0.088	0.268	-0.43 to 0.61	0.742
9.Suicidal thoughts or Wishes*	0.120	0.392	-0.24 to 1.79	0.480
10.Crying	0.029	0.226	0.89 to -0.41	0.226
11.Agitation	-0.375	0.307	-0.97 to 0.22	0.222
12.Loss of Interest	-0.472	0.301	-0.63 to 0.54	0.877
13. Indecisiveness	0.444	0.254	-0.05 to 0.90	0.081
14.Worthlessness*	0.697	0.308	0.09 to 1.83	0.024
15.Loss of Energy*	1.135	0.124	0.43 to 1.83	0.001
16. Changes in sleeping pattern	0.170	0.280	-0.38 to 0.72	0.547
17.Irritability	0.178	0.277	-0.36 to 0.72	0.520
18. Changes in appetite	0.269	0.251	-0.22 to 0.76	0.285
19. Concentration Difficulty	0.683	0.352	-0.009 to 1.37	0.653
20. Tiredness or fatigue*	0.714	0.339	0.04 to 1.37	0.035
21. Loss of Interest in Sex	0.235	0.212	-0.18 to 0.65	0.267
**Other EQ-5D Variables at baseline**				
Mobility*	2.617	0.573	1.49 to 3.73	0.001
Self-Care *	0.164	0.147	1.45 to 4.38	0.001
Activity*	1.764	0.422	0.93 to 0.63	0.001
Anxiety	0.164	0.580	-0.97 to 1.30	0.777

Table 4. Linear regression of possible variables predicting PHQ-9 depression at 3 months (p <0.1)				
**Predictor variable**	**β**	**Std Error**	**95% Confidence Interval**	**Sig p**
Constant	5.170	1.336	2.54 to 7.79	0.001
Pain group/no pain group allocation based on EQ-5D* (being in moderate to extreme pain)	-0.777	0.463	-1.68 to 0.13	0.094
Baseline EQ-5D Self-Care	1.063	0.600	-0.11 to 2.24	0.329
Baseline prescribed analgesics	-0.002	0.002	-0.007. to -0.02	0.272
Baseline EQ-5D Performing usual activity	0.272	0.401	-0.51 to 1.06	0.497
Type of health problem	-0.111	0.154	-0.41 to 0.19	0.473
BDI-II Question 9 "Suicidal thoughts or wishes"	0.712	0.406	-0.08 to 1.76	0.801
BDI-II Question 15 "Loss of energy"*	0.644	0.355	-0.55 to 1.13	0.071
Previous episodes of depression	0.044	0.115	-0.22 to 0.23	0.974
Baseline BDI-II Question 16 "Changes in sleeping pattern"	0.052	0.234	-0.40 to 0.51	0.825
Sex- (being male)*	-0.840	0.436	-1.69 to -0.15	0.054
Employment	0.142	0.099	-0.05 to 0.33	0.151
Baseline BDI Question 20 "Tiredness or fatigue"	0.013	0.326	-0.62 to 0.652	0.968
EQ-5D mobility at baseline*	1.760	0.635	0.51 to 3.00	0.006
Baseline PHQ-9 score*	0.480	0.047	0.38 to 0.573	0.000

Table 5. Regression model predicting PHQ-9 depression at 3 months. (p <0.05)				
**Predictor variable**	**β**	**Std Error**	**95% Confidence Interval**	**Sig p**
Constant	4.605	1.457	1.74 to 7.46	.002
Pain group/no pain group allocation based on EQ-5D* (being in moderate to extreme pain)	-1.011	0.507	-2.00 to -0.15	.047
EQ-5D mobility at baseline	1.760	0.635	0.51 to 3.00	.006
BDI-II q15Loss of energy at baseline	0.922	0.355	0.922 to 1.62	.010
Sex- (being male)	1.036	0.512	-2.04 to -0.32	.043
PHQ-9 score at baseline	0.480	0.047	0.38 to 0.57	.000

For peer review only
